# Fasting before living-kidney donation: effect on donor well-being and postoperative recovery: study protocol of a multicenter randomized controlled trial

**DOI:** 10.1186/s13063-021-05950-x

**Published:** 2022-01-06

**Authors:** C. A. J. Oudmaijer, R. C. Minnee, R. A. Pol, W. M. C. van den Boogaard, D. S. J. Komninos, J. van de Wetering, M. H. van Heugten, E. J. Hoorn, J. S. F. Sanders, J. H. J. Hoeijmakers, W. P. Vermeij, J. N. M. IJzermans

**Affiliations:** 1 Erasmus MC Transplant Institute, Department of Surgery, Division of Hepatobiliary and Transplantation Surgery, Erasmus University Medical Center, Dr. Molewaterplein 40, RG-220, 3015 GD Rotterdam, the Netherlands; 2 Princess Máxima Center for Pediatric Oncology, Utrecht, The Netherlands; 3 Oncode Institute, Utrecht, The Netherlands; 4 Department of Transplantation Surgery, University Medical Center Groningen, Groningen, the Netherlands; 5 Department of Internal Medicine, Division of Nephrology and Transplantation, Erasmus University Medical Center, Rotterdam, the Netherlands; 6 Department of Internal Medicine, Division of Nephrology and Transplantation, University Medical Center Groningen, Groningen, the Netherlands; 7 Erasmus MC Cancer Institute, Department of Molecular Genetics, Erasmus University Medical Center Rotterdam, Rotterdam, the Netherlands; 8 Institute for Genome Stability in Ageing and Disease, Medical Faculty, University of Cologne, Cologne, Germany; 9 Cologne Excellence Cluster for Cellular Stress Responses in Aging-Associated Diseases (CECAD), Centre for Molecular Medicine Cologne (CMMC), University of Cologne, Cologne, Germany

**Keywords:** Fasting, Living-kidney donation, Postoperative recovery, Ischemia-reperfusion injury

## Abstract

**Background:**

One of the main effectors on the quality of life of living-kidney donors is postoperative fatigue. Caloric restriction (CR) and short-term fasting (STF) are associated with improved fitness and increased resistance to acute stress. CR/STF increases the expression of cytoprotective genes, increases immunomodulation via increased anti-inflammatory cytokine production, and decreases the expression of pro-inflammatory markers. As such, nutritional preconditioning by CR or STF represents a non-invasive and cost-effective method that could mitigate the effects of acute surgery-induced stress and postoperative fatigue. To investigate whether preoperative STF contributes to a reduction in fatigue after living-kidney donation, a randomized clinical trial is indicated.

**Methods:**

We aim to determine whether 2.5 days of fasting reduces postoperative fatigue score in subjects undergoing living-kidney donation. In this randomized study, the intervention group will follow a preoperative fasting regime for 2.5 days with a low-dose laxative, while the control group will receive standard care. The main study endpoint is postoperative fatigue, 4 weeks after living-kidney donation. Secondary endpoints include the effect of preoperative fasting on postoperative hospital admission time, the feasibility of STF, and the postoperative recovery of donor and recipient kidney function. This study will provide us with knowledge of the feasibility of STF and confirm its effect on postoperative recovery.

**Discussion:**

Our study will provide clinically relevant information on the merits of caloric restriction for living-kidney donors and recipients. We expect to reduce the postoperative fatigue in living-kidney donors and improve the postoperative recovery of living-kidney recipients. It will provide evidence on the clinical merits and potential caveats of preoperative dietary interventions.

**Trial registration:**

Netherlands Trial Register NL9262. EudraCT 2020-005445-16. MEC Erasmus MC MEC-2020-0778. CCMO NL74623.078.21

**Supplementary Information:**

The online version contains supplementary material available at 10.1186/s13063-021-05950-x.

## Administrative information

**Table:** 

Title:	Fasting before living-kidney donation: effect on donor well-being and postoperative recovery
Acronym:	FAST-Study
Trial Registration:	NL74623.078.21, https://www.trialregister.nl/trial/NL9262
**WHO Trial Registration Data Set**	
Primary registry and trial-identifying number:	Netherlands Trial Register, NL9262
Date of registration in primary registry:	24-02-2021
Secondary identifying numbers:	EudraCT: 2020-005445-16
	MEC Erasmus MC: MEC-2020-0778
	CCMO: NL74623.078.21
Sources of monetary or material support:	-
Primary sponsor:	Erasmus MC Transplant Institute, the Netherlands
Secondary sponsor(s):	-
Contact for public & scientific queries:	Drs. C.A.J. Oudmaijer
	Erasmus MC Transplant Institute, University Medical Center Rotterdam
	Department of Surgery, Division of Hepatobiliary and Transplant Surgery
	Dr. Molewaterplein 40
	3015 GD Rotterdam
	E-mail: c.oudmaijer@erasmusmc.nl
	Tel: + 3110-7043541
Public title:	Fasting before living-kidney donation: effect on donor well-being and postoperative recovery
Scientific title:	Fasting before living-kidney donation: effect on donor well-being and postoperative recovery
Countries of recruitment:	The Netherlands
Health condition studied:	Living-kidney donation, ischemia-reperfusion-injury
Intervention:	Nutritional preconditioning by 60 hours preoperative fasting and the use of 1 daily dose of Macrogol 3350 (13.1 grams and electrolytes) for 3 days.
Key inclusion and exclusion criteria:	Adult patients and donors opting for living-kidney donation and transplantation at Erasmus MC, University Medical Center in Rotterdam, the Netherlands, and University Medical Center Groningen in Groningen, the Netherlands
	Inclusion criteria: age between 18 and 70 years old, BMI between 19 and 35 kg/m^2^, written informed consent and adequate understanding of the Dutch language
	Exclusion criteria: participants of the cross-over kidney donation program, participation in another prospective study for living-kidney donors, the use of double anticoagulants, the need for therapeutic anticoagulation during admission or a blood type or HLA-incompatible transplantation procedure.
Study type:	Multicentre, randomized controlled, parallel-group, superiority trial
Date of first enrolment:	01-06-2021
Target sample size:	180
Recruitment status:	Open for inclusion
Primary outcome:	Postoperative fatigue, scored by 36-Item Short Form Health Survey (RAND-36)
Key secondary outcomes:	Postoperative recovery of kidney function of the donor and recipient, incidence of delayed graft function and acute rejection in the transplanted patient, and adherence to the fasting regime
Protocol Version:	Version 2.7, verified on 09-08-2021
Funding	Investigator-initiated
Protocol Contributors:	Prof. dr. J.N.M. IJzermans Erasmus MC Transplant Institute University Medical Center Rotterdam Department of Surgery, Division of Hepatobiliary and Transplantation Surgery Principal investigator
	Dr. R.C. Minnee Erasmus MC Transplant Institute University Medical Center Rotterdam Department of Surgery, Division of Hepatobiliary and Transplantation Surgery Head Site Investigator
	Dr. R.A. Pol Department of Transplantation Surgery University Medical Center Groningen Head Site Investigator
	Dr. C.A.J. Oudmaijer Erasmus MC Transplant Institute University Medical Center Rotterdam Department of Surgery, Division Hepatobiliary and Transplantation Surgery Coordinating investigator
	Dr. J. van de Wetering Erasmus MC Transplant Institute University Medical Center Rotterdam Department of Internal Medicine Division of Nephrology and Transplantation Investigator, consulting nephrologist
	Prof. dr. J.H.J. Hoeijmakers Department of Molecular Genetics Erasmus MC Cancer Institute University Medical Centre Rotterdam & Princess Máxima Center for Pediatric Oncology Consulting Expert, biopsy/tissue research team
	Dr. Ing. W.P. Vermeij Princess Máxima Center for Pediatric Oncology Head of biopsy/tissue research team
All the abovementioned contributors have been extensively involved in study design.	
The inclusion of potential study subjects will be performed by C.A.J. Oudmaijer, R.C. Minnee, and R.A. Pol. The collection, management, analysis, and interpretation of the data will be performed by C.A.J. Oudmaijer. The writing of the report and decision to submit will be made by J.N.M. IJzermans, R.C. Minnee, R.A. Pol, C.A.J. Oudmaijer, W.P. Vermeij, J.H.J. Hoeijmakers, and J. van de Wetering.	
Contact information:	Drs. C.A.J. Oudmaijer Erasmus MC Transplant Institute University Medical Center Rotterdam Department of Surgery, Division of Hepatobiliary and Transplant Surgery
	Dr. Molewaterplein 40 3015 GD Rotterdam E-mail: c.oudmaijer@erasmusmc.nl Tel: +3110-7043541
Trial Sponsor:	Erasmus MC Transplant Institute, University Medical Center Rotterdam (Investigator-initiated study) Oncode Institute, Utrecht, The Netherlands
Role of Study Sponsor:	Oncode Institute provided support in preclinical research and indirectly in the writing of this manuscript. Oncode had no role in the design, preparation, or implementation of this trial.

## Background

Current postoperative care of most elective surgeries has been defined by enhanced recovery after surgery (ERAS) [1–3]. It achieves a quicker recovery after surgery, less fatigue, lower complication rates, and shorter hospital stay in comparison with former guidelines [1–5]. However, to date, no clear recommendation or evidence-based protocol of a preoperative diet has been developed. A preoperative diet employing caloric restriction (CR) might represent a non-invasive and cost-effective method that could mitigate postoperative fatigue and other short- and long-term consequences.

CR, also known as dietary restriction (DR), is defined as a reduced intake of calories, without causing malnutrition, and is associated with improved fitness and increased resistance to acute stress, at the expense of temporary attenuation of growth. In addition, it has been demonstrated to induce a lower risk of age-associated diseases and extended life span in pre-clinical studies [6–10]. In evolutionary terms, this response intends to promote survival during times of starvation and other types of stress, such as genotoxic stress caused by oxidative DNA damage; hence, we designated it “survival response” [11–14]. CR can be performed in different regimens, such as 20–40% reduced daily caloric intake for several weeks or longer, or as a more stringent and acute option: short-term fasting (STF), which could be performed right before a period of (surgery-induced) stress. Recent studies, mostly in animal models, showed preventive effects of CR and/or STF on genomic stress, ischemia-reperfusion injury (IRI), acute stress conditions, and aging [10, 15–22]. So far, the benefits of reducing caloric intake have partly been translated to humans: it has been proven feasible and safe in well-nourished patients before living-kidney donation [23–25] and was effective in improving radiological and pathological response during neoadjuvant chemotherapy treatment for breast cancer [26].

The upregulation of pathways involved in stress resistance suggests that CR and STF induce a protective state, resulting in increased expression of cytoprotective genes (e.g., NRF2-driven anti-oxidant response), enhanced immunomodulation via anti-inflammatory cytokine production, and decreased expression of pro-inflammatory markers [11, 21, 22, 27], all of which influence postoperative recovery. However, the activation of the protective pathways due to STF has not been extensively investigated in human subjects yet. STF has been proven beneficial in mitigating the effects of IRI [18, 20, 22, 28]. IRI is unavoidable during living-kidney transplantation and a risk factor for delayed graft function (DGF), acute rejection, and acute and chronic kidney injury, which results in increased morbidity and mortality of the transplanted patient [29–32]. Return of warm perfusion in ischemically damaged kidneys initiates a vicious cycle of events, initiated by the generation of reactive oxygen species due to activated xanthine oxidases, which damages cells and triggers an inflammatory response in turn involving the production of reactive oxygen species. This results in direct tissue damage and local and systemic, short- and long-term effects. For instance, it makes the graft more immunogenic by upregulating cytokines involved in the immune response [32, 33], thereby facilitating the development of DGF and acute rejection, inducing acute and chronic kidney injury [29–32]. As demonstrated in animal studies, the protective state induced by CR or STF might reduce the extent of the (generalized) inflammatory response [18, 20–22, 27, 28, 34].

STF represents a potential non-invasive and cost-effective method of mitigating the effects of acute surgery-induced stress and has only minor side effects. Previous studies mainly noted the feeling of hunger and constipation [23–25, 35]. Three recent pilot studies [23–25] showed no higher incidence of postoperative complications, compliance was high, and patients only reported minor discomfort due to constipation related to the preoperative diet.

Living-kidney donors are an excellent group to further investigate the effect of STF since they are in relatively healthy condition, the surgery is elective, and we aim to optimize the postoperative recovery of healthy subjects, undergoing a surgical procedure to help others. Previous research shows that long-term quality of life (QoL) after living-donor nephrectomy is excellent, while postoperative fatigue is a frequently mentioned effector on QoL, especially during the first 3 months after surgery [36, 37]. By reducing caloric intake through STF and thereby attenuating the systemic inflammatory response, we reasoned that postoperative fatigue that is especially notable during the first 3 months in living-kidney donors might be diminished. To investigate whether STF reduces postoperative fatigue after living-kidney donation, a randomized clinical trial is indicated.

The primary objective of this study is to determine the effect of STF in combination with a low-dose laxative on postoperative fatigue, 4 weeks after living-donor nephrectomy. Our secondary objectives include the effect of STF on postoperative admission time, physical activity, postoperative recovery of kidney function, molecular markers associated with the protective fasting state, the use of urinary extracellular vesicles (uEVs), and the feasibility and adherence to STF. To this end, we designed a multicenter, randomized controlled, parallel-group, superiority trial.

## Methods and analysis

The proposed study will be conducted at Erasmus MC Transplant Institute and University Medical Center Groningen (UMCG). Both hospitals are tertiary academic centers with extensive experience in living-kidney donation and transplantation and both have a broad experience in conducting clinical trials [https://www.transplantatiestichting.nl/files/2020-06/NTS_cijferbijlage_jaarverslag_2019.pdf]. We used the SPIRIT Checklist when writing our report [38]*.*

### Study population

Adult donors and patients opting for living-kidney donation and transplantation are eligible to participate in this study. Eligible donors must meet the following inclusion criteria: age 18–70 years, BMI 19–35 kg/m^2^, adequate understanding of the Dutch language, and the ability to provide written consent. An eligible donor who participates in the National Kidney Exchange program or in another prospective intervention trial will be excluded. The exclusion criteria applicable for potential recipients are the use of double anticoagulants, the need for therapeutic anticoagulation during admission, or a blood type or HLA-incompatible (positive crossmatch) transplantation procedure.

### Intervention

After study inclusion, the donor will be randomized to either the control or the intervention group. The intervention group will perform STF, starting 60 h before surgery. We selected the duration of the fasting diet based on previous research in animal models [18, 27] and other clinical trials [23–26]. Subjects can drink ad libitum water, tea, and coffee to maintain fluid balance, and they are allowed a maximum of 100 g of vegetables daily (cucumber, baby carrots, and baby tomatoes). To maintain electrolyte balance, they are allowed a max of 4 bouillon soups a day. After surgery, they can resume regular intake. To alleviate the symptoms associated with constipation during the fasting period, we added a low-dose laxative for 3 days: 1 dose of Macrogol 3350 daily. The use of laxatives could also affect the onset of the protective state induced by CR or STF by reducing the gastrointestinal transit time and therefore increase the STF effect. Details on the assigned intervention will be provided, both orally and in writing, by the study coordinator to the subject on both the day of randomization and the week before donation. The STF diet will be discontinued after review with the principal investigator if a subject experiences clinically significant side effects necessitating caloric intake, f.i. hypoglycemia. Any non-experimental treatments are allowed if it does not have any nutritional (caloric) value.

### Study procedures

Participants in our study will be subjected to an identical screening and work-up as non-participants, according to the local protocol. When there are no objections to kidney donation, the attending surgeon will decide which kidney is eligible. Surgery will be performed via minimally invasive procedures. Study protocols have been provided for both study locations, adjusted to local procedures.

Standardized questionnaires are used to analyze the influence of STF on postoperative fatigue after living-kidney donation. Questionnaires are provided digitally or on paper, depending on the preference of the participating donor. Questionnaires used in this study are the 36-Item Short Form Health Survey (RAND-36 [39, 40]) and the Quality of Recovery 40 (QoR-40 [41, 42]). The RAND-36 is one of the most widely used health-related quality of life survey instruments, comprising 36 items that assess eight health concepts. Both questionnaires have been validated as suitable instruments for assessing the postoperative quality of recovery [39–42]. These questionnaires will be completed at inclusion, 3 days before surgery, and 3 days and 4 and 12 weeks after surgery. All donors included in this study will be given an accelerometer (GENEActiv [43–45]) which will be worn 24 h a day from 1 week before up and until 2 weeks after surgery. Tissue biopsies on both kidney and excess tissue will be acquired during both the donor nephrectomy and kidney transplantation (Additional file 1).

Transplant recipients will be submitted to an identical preoperative screening, work-up, surgery, and postoperative care as non-study participants, according to the local protocol. To monitor the effects of STF on the kidney, uEVs will be isolated and analyzed from the urine produced by the transplanted kidney during implantation to identify non-invasive biomarkers (further described in Additional file 1). An overview of procedures is provided for the donor (Additional file 1: Table S1) and recipient (Additional file 1: Table S2).

#### Center-specific procedures

At Erasmus MC, uEVs will be analyzed in additional urine samples collected from both the donor and recipient. In total, four additional samples will be collected (see Additional file 1 and its Fig. S1).

### Outcomes

The primary outcome is postoperative fatigue after living-kidney donation, scored by RAND-36 [39, 40]. A higher score represents a higher QoL. RAND-36 will be scored at set time points, and the primary endpoint will be measured 4 weeks after surgery. The absolute difference in the RAND-36 score (“physical functioning”, “energy/fatigue,” and the aggregate score) will be compared between both treatment arms. Reference data on postoperative fatigue after living-kidney donation is available from Klop et al. [36]. Literature on the RAND-36 shows that small differences could be interpreted as clinically important [46].

Our secondary endpoints comprise several aspects of the preoperative fasting diet, postoperative recovery, and IRI, as listed below. All the secondary outcomes will be compared between the study arms to identify an effect due to the study intervention.

– Short-term postoperative recovery will be measured using the QoR-40 [41, 42]. The QoR-40 will be scored on set time intervals during our study runtime (see Table S1).

– Postoperative hospital admission time will be measured in days since surgery.

– Adherence to the fasting regime will be measured in several ways: by self-reported adherence at hospital admission, by change in body weight during the fasting period, and by blood samples drawn on the day of surgery. Blood samples will be used for several laboratory measurements: e.g., fasting glucose (mmol/l), fasting insulin (pmol/l), albumin (g/l), urea (mmol/l), creatinine (μmol/l), cystatin-C (mg/l), ferritin (μg/l), HbA1c (mmol/mol), and total cholesterol (mmol/l). These measurements will be performed by our centers’ clinical chemistry departments, according to standard procedures.

– Physical activity before, during, and after hospital stay will be determined by an accelerometer (GENEActiv [43–45]). Study subjects in any treatment arm will wear this accelerometer from 1 week prior to 2 weeks after surgery. Open-source software will be used to monitor physical activity during the pre- and postoperative periods.

– Laboratory measurements, assessed by routine lab procedures in blood samples, including creatinine (μmol/l), cystatin-C (mg/l), urea (mmol/l), albumin (g/l), and eGFR calculated via CKD-EPI, will be used to determine the postoperative kidney function of the donor and the recipient.

– The incidence of DGF (defined as the need for dialysis within 1 week after transplantation) and functional-DGF (failure of serum creatinine to decrease by at least 10% daily on 3 consecutive days during the first week post-transplant) will be assessed. The diagnosis of (f-)DGF will be determined on data extracted from the electronic health record.

– The incidence of acute rejection, diagnosed by a kidney biopsy with histological findings in accordance with the Banff classification [47], will be assessed in the recipients of both treatment arms. The diagnosis will be extracted from the electronic health record.

– Blood samples and tissue biopsies acquired on the day of surgery will be used to determine the upregulation of cytoprotective genes and anti-inflammatory markers associated with the fasting state. Additional file 1 provides extensive information regarding the histological and molecular analysis. In general, the samples will be used to conduct an expression analysis of genes responsive to fasting, such as cytoprotective antioxidant genes and GH/IGF1 markers and DNA damage and toxicity markers (e.g., yH2AX signal, SS/SH redox state, and inflammatory markers).

### Sample size calculation

Previously conducted questionnaires (RAND-36) in our cohort of living-kidney donors show a mean physical functioning on 4 weeks after surgery of 72 ± 18.4 (mean ± SD). Using G*Power v3.1.9.7, we calculated that we need 162 subjects to find a statistically significant (*p* < 0.05), 15% improvement in postoperative fatigue with a power of 0.95, measured by the RAND-36 (score of 82.5, effect size 0.57). To account for the possible drop-out of study participants, estimated at ± 10%, the total sample size was set at 180 subjects. These 180 subjects will be randomized into 2 groups of 90 subjects. We aim to include 90 patients per study center.

### Recruitment

According to the annual report of the Dutch Transplant Foundation, a total of 501 living-kidney donors donated in 2019, of which 100 donated at Erasmus MC and 83 at UMCG [38]. This sums up to 183 living-kidney donations in 2019. Therefore, we consider it feasible to include all our participants in 2 years, depending on the extent of the current COVID-19 pandemic. Eligible donors and recipients visiting the outpatient clinic will be identified and invited to participate in the study. The consulting physician informs the eligible participant about the general outline of the trial. If eligible donors and recipients are interested in participation, the study investigator explains the study design, purpose, and intervention in more detail. Subjects will then receive additional written information about the study, after which they are offered a reflection period. Finally, they are asked to sign the informed consent during their next visit to the outpatient clinic.

### Randomization

The computer program Castor EDC (Amsterdam, The Netherlands), used in compliance with laws and regulations, will be used to centrally randomize study subjects into the study arms after acquiring informed consent. Computerized stratified block randomization will be performed to determine the allocation to a treatment group, and blocks sized 4, 6, and 8 will be used. Participants will be randomized in a ratio of 1:1, and stratification will be employed for the study center and subject sex. Participant study number and the result of randomization will be provided immediately to all parties who should receive such notifications. Trial participants and care providers cannot be blinded to the result of randomization, since there is no placebo for STF. Enrollment and randomization will be performed in both study centers by trained medical personnel with adequate knowledge of the study procedures, the ability to provide adequate instructions concerning the fasting intervention, and the experience in conducting clinical trials.

### Data collection and management

All study data will be collected from the electronic health system by trained medical personnel. Each study subject has a personal electronical case report form, of which a blank copy can be supplied on request. Due to the nature of our study population, relatively healthy participants who typically are motivated to participate in clinical research, we expect relatively high participant retention and complete follow-up. If a subject refrains from further participation in the trial, we ask permission to collect the results from the standard of care procedures.

All relevant study data will be stored in Castor EDC, in compliance with the Dutch and international laws and regulations. Data entry will be conducted by trained medical personnel. Data will be range-checked during data entry to further enhance the data quality. A data management plan has been constructed in accordance with the academic hospital procedures. No personal documents will be listed, and each subject is pseudonymized via study number, listed on all study-related documentation. All data will be handled confidentially in compliance with the EU General Data Protection Regulation and will be confidentially saved for a maximum of 20 years after the completion of the study, in compliance with the Dutch laws and regulations.

### Statistical analysis

Statistical analysis will be performed using the IBM SPSS software version 25.0 (Chicago, IL) or R version 4.0.3 or newer. An intention-to-treat analysis will be conducted to account for the feasibility of STF. A two-sided significance level of 0.05 will be used for all primary and secondary analyses unless stated otherwise. An analysis will be performed with multiple imputation in the case of missing data. There will be no interim analysis due to the relatively low number of patients needed and the patients included are healthy, mentally capable, non-critical, and are not incapacitated.

Also, the short-term fasting diet used in this study is not known to harm the involved patients.

To assess the difference between the intervention and control group for the primary endpoint, we will construct a multivariable linear regression model adjusted for confounders such as age and baseline RAND-36 score. The statistically significant change in postoperative fatigue due to the intervention will be assessed using a Wald test*.*

In the secondary analysis, the multivariable linear regression model analysis from the primary analysis will be performed, including compliance to the fasting diet as an independent variable.

The difference between the study groups in postoperative fatigue, measured by QoR-40, will be analyzed using a multivariable linear regression model adjusted for relevant confounders, such as age and baseline QoR-40 score. An additional analysis will be performed including compliance to the diet as an independent variable. The difference in hospital admission time will be assessed by a multivariable linear regression adjusted for relevant confounders. Adherence to STF will be analyzed via descriptive statistics. Body weight will be compared between the two groups by a mixed model analysis; the independent variables in this model will f.i. include age, sex, and an interaction term between time and study group.

A mixed model will analyze the difference between the two groups in pre-, per-, and postoperative physical activity. Relevant confounders such as age and sex will be included, and an interaction term between time and group will be incorporated.

A mixed model will be constructed to investigate the effect of preoperative fasting on postoperative kidney function. This model will be computed separately for both the transplanted patients and the donors. Confounders such as age, sex, preoperative kidney function, and compliance to the diet will be incorporated in the model, as well as an interaction term between time and group. The incidence of DGF, functional-DGF, and acute rejection will be calculated as a proportion and compared by computing a multivariable logistic regression analysis.

### Data monitoring

We classified the risk of our study as negligible. As described by the Erasmus MC METC monitoring plan (version February 2013), we will have a monitoring frequency of once per year. Monitoring activities are briefly described as follows: confirming that the Trial Master File and Investigator Site Files are present and complete, confirming that the study staff is adequately instructed on the study procedures, assessment of patient inclusion rate, consent, compliance, source document verification, and verification of accessibility of study procedures.

### Potential harms

Adverse and serious adverse events will be registered as required by the regulations stated by the medical ethical committee of Erasmus MC*.* Adverse events have been defined as any undesirable experience occurring to a subject during the study, whether or not considered related to the fasting diet. All adverse events reported spontaneously by the subject or observed by the investigator or the staff will be recorded, unless these adverse events are part of the normal postoperative time period and are not signs or symptoms of serious postoperative complications (Clavien-Dindo class ≥ 3) [48]. A serious adverse event, defined according to the definition of the CCMO, is only regarded as related to the study if it occurs within 3 months after the study intervention and surgery. Prolonged hospitalization of the elective surgery will not be considered an adverse event unless it derives from serious postoperative complications (Clavien-Dindo class ≥ 3) [48].

Based on the hypothesis that STF induces beneficial effects, the intervention group may experience better subjective well-being and less fatigue as compared to those in the control group. Three recent clinical pilot studies [23–25] investigating the risks and feasibility of preoperative CR showed no higher incidence of complications and patients only reported minor discomfort during the preoperative diet. Therefore, we do not expect any potential issues concerning patient safety or well-being. Nevertheless, precautions will be taken to ensure that patients who are in the fasting group will maintain fluid balance without consuming calories. Participants will receive extensive practical information concerning the diet. Potential compensation for any participant who suffers harm due to trial participation has been covered by the academic hospital insurance, in accordance with the Dutch laws and regulations. Since the STF diet is related to the surgery conducted, participants cannot continue this specific fasting diet after completing the study. However, adhering to a fasting or calorie-restricted diet can be done on an individual basis, if preferred and in consensus with healthcare providers.

The frequency and procedures of auditing will not be changed for our study. Regular auditing, the standard for this academic hospital, will continue as normal. This process is done independently from the study investigators.

### Ethics and dissemination

The medical ethical committee of Erasmus MC has approved the study protocol, patient information files, consent procedures, and other study-related documents and procedures. This medical committee is an extension of the CCMO*.* The trial has been registered under EudraCT 2020-005445-16 and medical ethical assessment numbers MEC-2020-0778 and NL74623.078.21.

The procedure regarding study amendments has been defined in the study protocol. A “substantial amendment” is defined as an amendment that is likely to affect to a significant degree: the safety or physical or mental integrity of the subjects of the trial, the scientific value of the trial, the conduct or management of the trial, or the quality or safety of any intervention used in the trial. Substantial amendments will be submitted to the medical ethical committee and will only be conducted after approval. If a protocol amendment has been accepted, it will be changed accordingly in the trial registry and any publications. Informed consent will be obtained in accordance with relevant laws and regulations (e.g., ICH-GCP) by qualified study researchers. Due to the nature of this study, no information file or procedure has been defined for surrogates. An additional consent will be provided for the use of participant data and biological specimens in related studies in the future; this separate consent is not required to participate in this study. A model consent form has been provided in Additional file 2.

### Confidentiality

Upon study inclusion, each subject will be assigned a study number which will be listed on all study-related documentation. Data entry will be conducted in the pseudonymized form with study numbers. The principal investigator will keep a subject identification log, unique per study center, which holds the record of the personal identification data linked to each study number. This record is filed at the investigational site and can only be accessed by the investigator and the supporting site staff. Research data that can be traced to individual persons can only be viewed by authorized personnel. The principal investigator is responsible for the final trial dataset. Research data can only be viewed by authorized personnel, which encompasses members of the research team, members of the healthcare inspection, and study monitors. Potential data sharing requests will be approved by a representative of the academic hospital and the principal investigators.

## Discussion

This study aims to investigate the effect of STF in combination with a low-dose laxative on postoperative fatigue, 4 weeks after living-donor nephrectomy. Living-kidney donors are an excellent group to further investigate the effect of STF, and we aim to optimize the postoperative recovery of healthy subjects, undergoing a surgical procedure to help others.

### Dissemination of study findings

The principal investigator is responsible for the public disclosure and publication of the research data. The (final) results of the study will be summarized in a report/article and will be submitted for publication in a medical journal. Also, all participating subjects or their family will receive a layman’s summary of the (final) results of the study if they so desire. The trial has also been registered in a Dutch public trial registration registry, available at http://www.trialregister.nl/trialreg/index.asp.

Eligibility for authorship has been defined by the ICMJE guidelines for the Role of Authors and Contributors (http://www.icmje.org/recommendations/browse/roles-and-responsibilities/defining-the-role-of-authors-and-contributors.html). The full protocol, anonymized dataset, and statistical code can be granted after submitting a request with the principal investigator and the institution’s technology transfer office.

## Trial status

This trial is currently ongoing and actively recruiting participants (23/180). It has a planned duration of 2 years, with recruitment having started in May 2021 and continuing to May 2023. All changes in the study protocol will be recorded in the clinical trials registry (Netherlands Trial Register: NL9262).

## Supplementary Information

**Additional file 1:.** Figures and biological specimens.

**Additional file 2:.** Informed consent forms.

**Additional file 3:.** Rebuttal letter.

## Data Availability

Not applicable.

## Abbreviations

ERAS: Enhanced recovery after surgery
CR: Caloric restriction
DR: Dietary restriction
STF: Short-term fasting
IRI: Ischemia-reperfusion injury
DGF: Delayed graft function
QoL: Quality of life
uEVs: Urinary extracellular vesicles
UMCG: University Medical Center Groningen
RAND-36: Short Form Health Survey
QoR-40: Quality of Recovery 40
