# Supplementary material for: Fasting before living-kidney donation: effect on donor well-being and postoperative recovery: study protocol of a multicenter randomized controlled trial
Source: Trials. 2022 Jan 6;23:18. doi: 10.1186/s13063-021-05950-x (PMC8733810; doi:10.1186/s13063-021-05950-x)
Supplement: Supplementary file 1 — Additional file 1:. Figures and biological specimens. [file 13063_2021_5950_MOESM1_ESM.docx]

**Appendix A: Study Workflow**

**Overview of Study Procedures Donor**

Table 1: Study overview / timing of measurements, Donor

| **Study Moment (0 = Surgery)** | **T = -4** | **T = -3** | **T = -2** | **T = -1** | **Surgery**  **T = 0** | **T = +1** | **T = +2** | **T = +3** | **T = +4** | **T = +5** | **T = +6** | **T = +7** |
| --- | --- | --- | --- | --- | --- | --- | --- | --- | --- | --- | --- | --- |
| Study Moment Timing, POD^1^ | **Identification** | **-12 weeks^2^** | **-1 week** | **POD -3** | **POD 0** | **POD 1** | **POD 2** | **POD 3** | **POD 4** | **+ 2 weeks** | **+4 weeks** | **+12 weeks** |
| OC: Outpatient Clinic / HA: Hospital / H: Home | **OC** | **OC** | **H** | **H** | **HA** | **HA** | **HA** | **HA** | **HA** | **H** | **OC** | **OC** |
| **Study Information** | **X** | **-** | **-** | **-** | **-** | **-** | **-** | **-** | **-** | **-** | **-** | **-** |
| **Informed Consent, Inclusion & Randomisation** | **-** | **X** | **-** | **-** | **-** | **-** | **-** | **-** | **-** | **-** | **-** | **-** |
| **Measurements** |  |  |  |  |  |  |  |  |  |  |  |  |
| Weight/Height | **-** | **X** | **-** | **-** | **X** | **X** | **X** | **X** | ***-*** | ***-*** | **X** | **X** |
| Quality of Life (RAND-36 & QoR-40) | **-** | **X** | **-** | **X** | **-** | **-** | **-** | **X** | **-** | **-** | **X** | **X** |
| Smartwatch / Activity Tracker | **-** | **-** | **X** | **🡪 🡪 ^3^** | **🡪 🡪** | **🡪 🡪** | **🡪 🡪** | **🡪 🡪** | **🡪 🡪** | **X** | **-** | **-** |
| **Laboratory testing** | **-** |  |  |  |  |  |  |  |  |  |  |  |
| - Blood | **-** | **X** | **-** | **-** | **X** | **X** | **X** | **X** | **-** | **-** | **X** | **X** |
| - Urine | **-** | **-** | **-** | **-** | **X** | **-** | **-** | **-** | **-** | **-** | **-** | **-** |
| **Biopsies** |  |  |  |  |  |  |  |  |  |  |  |  |
| - Ureter | **-** | **-** | **-** | **-** | **X** | **-** | **-** | **-** | **-** | **-** | **-** | **-** |
| - Kidney Biopsy 1 & 2 | **-** | **-** | **-** | **-** | **X** | **-** | **-** | **-** | **-** | ***-*** | **-** | **-** |
| - Perirenal fat | **-** | **-** | **-** | **-** | **X** | **-** | **-** | **-** | **-** | **-** | **-** | **-** |

^1^ POD = Post-Operative Day

^2^ Can vary due to the planned date of surgery

^3^ 🡪🡪 Meaning continuous measurement.

**Overview of Study Procedures Recipient**

Table 2: Study overview / timing of measurements, Recipient

| **Study Moment (0 = Surgery)** | **T = -2** | **T = -1** | **Surgery**  **T = 0** | **T = +1** | **T = +2** | **T = +3** | **T = +4** | **T = +5** | **T = +6** | **T = +7** | **T = +8** | **T = +9** |
| --- | --- | --- | --- | --- | --- | --- | --- | --- | --- | --- | --- | --- |
| Study Moment Timing, POD^1^ | **Identification** | **-12 weeks^2^** | **POD 0** | **POD 1** | **POD 2** | **POD 3** | **POD 4** | **POD 5** | **POD 6** | **POD 7** | **+4 weeks** | **+12 weeks** |
| OC: Outpatient Clinic / HA: Hospital / H: Home | **OC** | **OC** | **HA** | **HA** | **HA** | **HA** | **HA** | **HA** | **HA** | **HA** | **OC** | **OC** |
| **Study Information** | **X** | **-** | **-** | **-** | **-** | **-** | **-** | **-** | **-** | **-** | **-** | **-** |
| **Informed Consent, Inclusion** | **-** | **X** | **-** | **-** | **-** | **-** | **-** | **-** | **-** | **-** | **-** | **-** |
| **Laboratory testing^3^** |  |  |  |  |  |  |  |  |  |  |  |  |
| - Blood | **-** | **X** | **X** | **X** | **X** | **X** | **X** | **X** | **X** | **X** | **X** | **X** |
| - Urine | **-** | **-** | **X** | **-** | **-** | **-** | **-** | **-** | **-** | **-** | **-** | **-** |

^1^ POD = Post-Operative Day

^2^ Can vary due to the planned date of surgery

^3^ All labarotory testing in the recipient is standard of care, no additional invasive procedures are necessary.

**Appendix B: biological specimens**

**Overview of biological specimens**

- Kidney biopsy:
  - Biopsy 1 Acquired after induction of cold ischemia
  - Biopsy 2 Acquired 10-15 minutes after reperfusion

Size: 2 by 2 mm

- Surplus tissue, distal Ureter:
  - Proximal sub-part Acquired 10-15 minutes after reperfusion
  - Middle sub-part Acquired after induction of cold ischemia
  - Distal sub-part Acquired after induction of cold ischemia

Size: 10-15 mm

- - Perirenal fat Acquired after induction of cold ischemia

Size: 20 by 20 mm

- Blood samples: Preoperative Donor 6 mL
- Urine samples:
  - Preoperative Donor 50 mL
  - Preoperative Recipient 50 mL
  - Urine produced during surgery 5-50 mL
  - Postoperative Donor 50 mL
  - Postoperative Recipient 50 mL

**Tissue and blood samples**

Acquirement of samples

To obtain markers of the protective stress response and the impact of ischemia/reperfusion injury, two kidney biopsies are performed during the surgery. The first kidney biopsy is taken just after removal of the graft from the donor, the second biopsy will be taken 15 minutes after reperfusion of the kidney in the recipient. The samples will be acquired via a standardized procedure to exclude any possible variations between participating locations. Both biopsies will be sized 2 mm by 2 mm.

Surplus tissue, perirenal fat and part of distal ureter, can easily be acquired since there is a surplus during surgery, which would normally be discarded. The distal biopsies of the ureter will be acquired twice during surgery. The first two biopsies (distal sub-part and middle sub-part) are taken just after removal of the graft from the donor, the third biopsy (proximal sub-part) will be taken 10-15 minutes after reperfusion. Special care will be taken to not significantly shorten the length of the ureter to be transplanted.

Kidney biopsy during surgery is a relatively safe procedure [1, 2]. The main complication being post-procedure bleeding with an incidence of 1.2% [1-6]. Prior to obtaining the biopsy, the surgeon will have a clear view of the kidney during the procedure, will have time to anticipate on the situation, and the ability to acquire haemostasis if needed. At the end of the surgery, the kidney is inspected to determine whether adequate haemostasis has been achieved.

The aforementioned kidney- and ureter biopsies, taken after induction of cold ischemia during donor surgery, are used to determine critical parameters of induction and the presence of the ‘survival response’. In previous studies, we determined a panel of cytoprotective genes activated during fasting regimens [7, 8]. These include elevation of antioxidant genes and suppression of the GH/IGF1 axis, and this will be determined via RNA expression analysis. The fat tissue, from surplus available around the surgical area, will be analysed using metabolomics.

Following transplantation and subsequent ischemia-reperfusion injury, a second small kidney biopsy and surplus piece of ureter will be used to measure tissue damage parameters indicating possible protection due to fasting. Part of the ureter that will be excised at the end of the donor surgery will be snap-frozen and part will be fixed in formaldehyde for immuno-histochemistry to determine for example DNA damage parameters or oxidative stress signalling. These include overall thiol (SS/SH) oxidation state analysed by redox western blots, DNA damage levels using γH2AX foci analysis, and inflammatory state using gene expression analysis.

Analysis of blood & tissue samples

*CR/Fasting + damage markers*

GH, GHR, IGF-I, DIO1, DIO3, Leptin, Ghrelin, IGFBPs, ACOT1, RRM1, EGR1, IGF-1 receptor, FGF-21, GDF-15, TRIM24, SIRT1, ENT1, NRF2, HO-1, PGC-1a, ELOVL3, p16, p21, p53, IL-6, TNFa

Total RNA will be isolated from tissue specimens using Trizol for analysis of CR/Fasting and DNA damage response markers using qPCR or RNA-sequencing. Expression data of markers will be correlated to blood values obtained from the diagnostic laboratory. Where possible protein levels will be assessed using western blotting.

*Immunohistochemistry/Immunofluorescence*

Senescence: HMGB1, lamin B1, p21

DNA damage: yH2AX, 53BP1, RAD51

Apoptosis: TUNEL, cleaved caspase-3

*Redox western blot*

Assay for monitoring thiol redox states, which are known to change upon dietary restriction/fasting. With use of chemical modifiers that can specifically bind covalently with free thiol groups, thereby inactivating them. Reducing agents cannot remove these modifications. After subsequent reduction of oxidized thiol groups, which were engaged in disulfide bonds, these could be labelled with a second compound. These can then be analysed using mass spectrometry, western blotting, or histology [7, 9, 10].

*Organotypic slices*

This method has been described for several tissue types [11-13], but generally is as follows: tissue samples should be obtained as quick as possible but no later than 3 hours after surgical resection and kept at 4°C during transport to the laboratory. Keep tissue sample in culture medium, on ice while transported. If required, remove excess fat from tissue sample and make slices using a vibratome (Leica). Thickness of slices are determined in advance, ideally 200-300 µm. Slices should be transferred to wells plate/culture dish containing culture medium and subsequently incubated at 37°C, 5% CO2, on a shaking platform (*60 rpm*). Additional assays (e.g. EU/EdU incubation) can be performed while slices are in culture. Tissue slices can be fixed in paraformaldehyde or formalin, embedded in paraffin and sliced in 4 µm sections using a microtome. Material can then be used for immunohistochemistry or immunofluorescence.

**Urinary extracellular vesicles**

Background

To monitor the effects of CR on the kidney, a non-invasive biomarker would be desirable. To this end, the emerging field of urinary extracellular vesicles (uEVs) is of interest. uEVs are secreted by kidney epithelial cells and can be isolated from the urine. uEVs contain DNA, RNA, and protein with biomarkers relevant for kidney function in health and disease [14]. The connection between IRI, local inflammatory signalling and dietary interventions is complex. In IRI and diabetic nephropathy models, tubular epithelial cells develop senescence with a chronic senescence-associated secretory phenotype, including inflammation and fibrosis-inducing signalling [15, 16]. The possibility to detect these processes in uEVs was shown by Asvapromtada et al. [17], who observed that after experimentally induced IRI, rats show both altered uEV cargo and absolute uEV abundance. Other responses in acute kidney injury have been identified and the influence of systemic serum glucose on uEV content was already shown in hyperglycaemic patients with diabetes [17-23]. So far, very few studies have performed direct comparison between uEVs and tissue analyses in humans. Specifically, in kidney transplantation, uEVs isolated during transplantation have been correlated with DGF and in further assessments biomarkers predictive of future graft function have been found [19, 24, 25].

Objectives

The urine samples collected on the day of surgery will be used to investigate the use of uEV to non-invasively characterize the effects of kidney transplantation in human subjects and to further determine the effect of fasting by studying uEV-contained and associated biomarkers. We will compare the change in uEV biomarkers between fasting and non-fasting subjects, as well as how these change during and after transplantation, investigate the correlation between uEV-contained and -associated biomarkers, tissue histopathological changes and post-operative kidney function. This will further define the use of uEVs as a source of predictive biomarkers for future graft function.

Analysis of urine samples

EMC samples and samples received from associated centres will be stored and analysed in sets of 12 when equal numbers of fasting and non-fasting individuals are available. This is important to prevent potential batch effects introduced by variations in isolation efficiency.

Briefly, uEV isolation will require urine samples to be thawed, after which a protease and RNAse inhibitor will be added. The samples will be centrifuged at 3000 RCF for 10 minutes to remove large contaminants. The derived supernatant will then be transferred to ultracentrifuge containers before processing at 17.000 RCF for 20 minutes to pellet aggregated uEV and suspended cells. Supernatant from this step will be saved, and the pelleted material resuspended and treated with DTT to dissolve the uromodulin mesh.

After DTT treatment the dissolved pellets will again be processed at 17.000 RCF for 20 minutes to remove remaining cells and non-uEV material. The derived supernatant will be combined with the supernatant from the first 17.000 RCF step for definitive uEV isolation. The combined supernatants will be processed at 180.000 to 200.000 RCF depending on the starting volume and therewith required rotor, centrifuged for two hours. The final supernatant will be discarded, and the pellet resuspended in 150 µL PBS for protein or RNA quantification.

This whole ultracentrifuge protocol takes place in a single day for 6-12 samples at a time, and the resulting extracellular vesicle isolates can be stored at -80°C for any required period of time until protein or RNA quantification can be performed.

Statistical Analysis

Changes in uEV biomarkers will be compared between study arms as well as between sample collection time points. Depending on the exact groups being compared, the bioinformatics approach will be different, but in general Q-values will be calculated per protein or RNA, and significant results weighted by their fold-change. Subsequently, the identified discriminating biomarkers will be used to identify pathways with changed activity between groups. This not only allows for the identification of both predictive and representative biomarkers in general, but it also reveals the pathways altered by fasting, ischemia-reperfusion, and associated with graft function after transplantation.


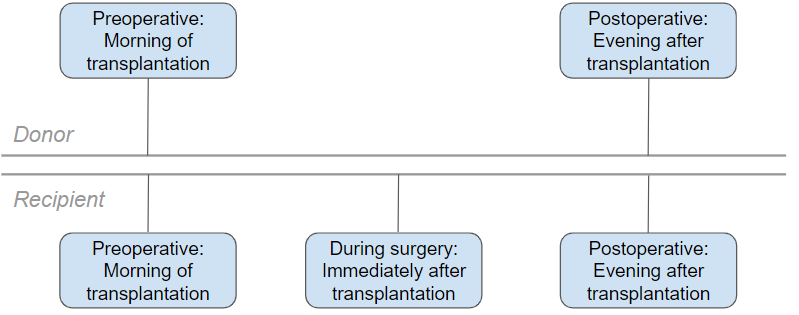
 Figure 1. Overview of timing of acquirement of samples


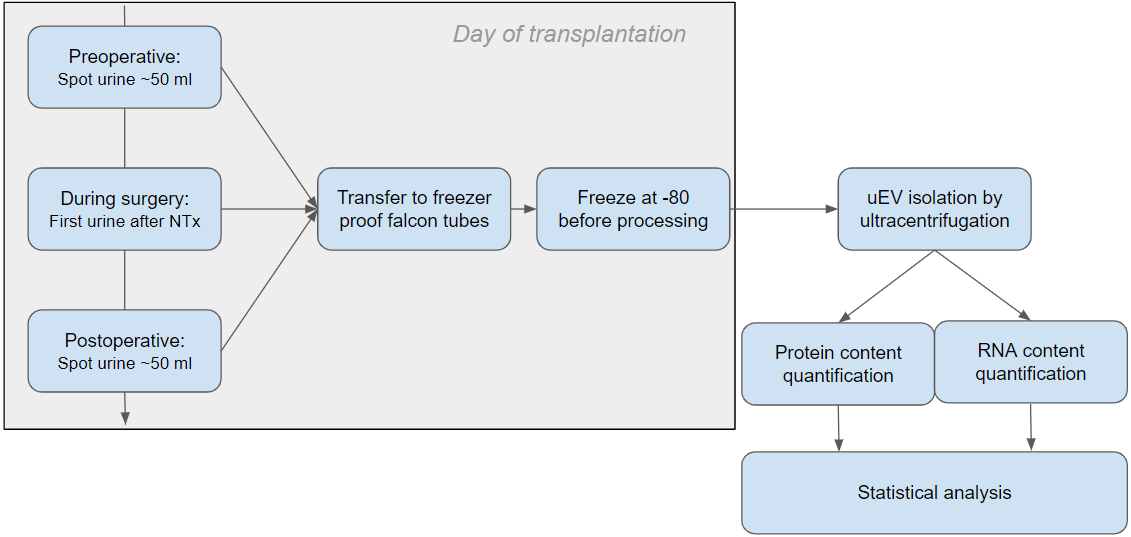
Figure 2: Example workflow for recipient samples

Figure 3: Example workflow for donor samples


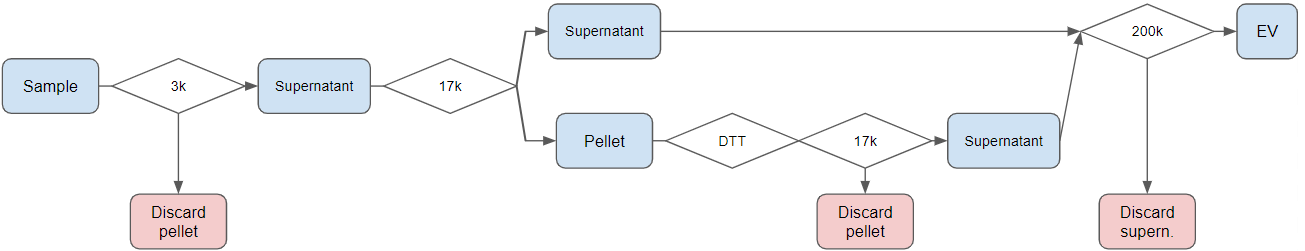


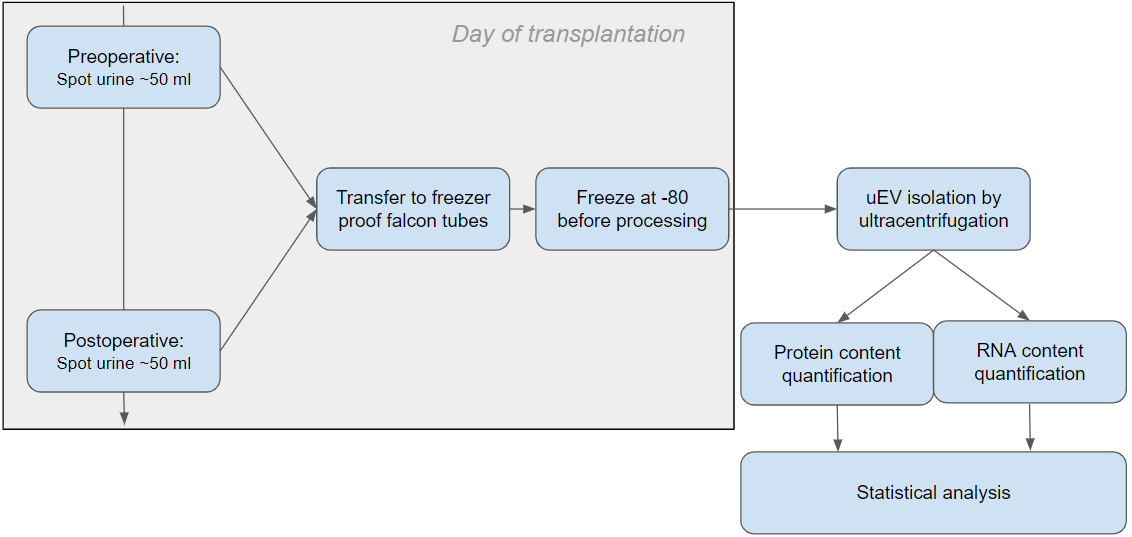
Figure 4: Extracellular vesicle isolation protocol

**References**

1. Gimenez LF, Micali S, Chen RN, Moore RG, Kavoussi LR, Scheel PJ, Jr. Laparoscopic renal biopsy. Kidney Int. 1998;54(2):525-9.

2. Shetye KR, Kavoussi LR, Ramakumar S, Fugita OE, Jarrett TW. Laparoscopic renal biopsy: a 9-year experience. BJU Int. 2003;91(9):817-20.

3. Madaio MP. Renal biopsy. Kidney Int. 1990;38(3):529-43.

4. Corapi KM, Chen JL, Balk EM, Gordon CE. Bleeding complications of native kidney biopsy: a systematic review and meta-analysis. Am J Kidney Dis. 2012;60(1):62-73.

5. Redfield RR, McCune KR, Rao A, Sadowski E, Hanson M, Kolterman AJ, et al. Nature, timing, and severity of complications from ultrasound-guided percutaneous renal transplant biopsy. Transpl Int. 2016;29(2):167-72.

6. Whittier WL, Korbet SM. Timing of complications in percutaneous renal biopsy. J Am Soc Nephrol. 2004;15(1):142-7.

7. Schumacher B, van der Pluijm I, Moorhouse MJ, Kosteas T, Robinson AR, Suh Y, et al. Delayed and accelerated aging share common longevity assurance mechanisms. PLoS genetics. 2008;4(8):e1000161.

8. Jongbloed F, de Bruin RWF, Steeg HV, Beekhof P, Wackers P, Hesselink DA, et al. Protein and calorie restriction may improve outcomes in living kidney donors and kidney transplant recipients. Aging (Albany NY). 2020;12.

9. Rudyk O, Eaton P. Biochemical methods for monitoring protein thiol redox states in biological systems. Redox Biol. 2014;2:803-13.

10. La Fata G, van Vliet N, Barnhoorn S, Brandt RMC, Etheve S, Chenal E, et al. Vitamin E Supplementation Reduces Cellular Loss in the Brain of a Premature Aging Mouse Model. The journal of prevention of Alzheimer's disease. 2017;4(4):226-35.

11. Schwarz N, Hedrich UBS, Schwarz H, P AH, Dammeier N, Auffenberg E, et al. Human Cerebrospinal fluid promotes long-term neuronal viability and network function in human neocortical organotypic brain slice cultures. Sci Rep. 2017;7(1):12249.

12. Naipal KA, Verkaik NS, Ameziane N, van Deurzen CH, Ter Brugge P, Meijers M, et al. Functional ex vivo assay to select homologous recombination-deficient breast tumors for PARP inhibitor treatment. Clin Cancer Res. 2014;20(18):4816-26.

13. Linsley JW, Tripathi A, Epstein I, Schmunk G, Mount E, Campioni M, et al. Automated four-dimensional long term imaging enables single cell tracking within organotypic brain slices to study neurodevelopment and degeneration. Commun Biol. 2019;2:155.

14. van Niel G, D'Angelo G, Raposo G. Shedding light on the cell biology of extracellular vesicles. Nature reviews Molecular cell biology. 2018;19(4):213-28.

15. Korolchuk VI, Miwa S, Carroll B, von Zglinicki T. Mitochondria in Cell Senescence: Is Mitophagy the Weakest Link? EBioMedicine. 2017;21:7-13.

16. Samarakoon R, Helo S, Dobberfuhl AD, Khakoo NS, Falke L, Overstreet JM, et al. Loss of tumour suppressor PTEN expression in renal injury initiates SMAD3- and p53-dependent fibrotic responses. The Journal of pathology. 2015;236(4):421-32.

17. Asvapromtada S, Sonoda H, Kinouchi M, Oshikawa S, Takahashi S, Hoshino Y, et al. Characterization of urinary exosomal release of aquaporin-1 and -2 after renal ischemia-reperfusion in rats. Am J Physiol Renal Physiol. 2018;314(4):F584-f601.

18. Oshikawa S, Sonoda H, Ikeda M. Aquaporins in Urinary Extracellular Vesicles (Exosomes). International journal of molecular sciences. 2016;17(6).

19. Sonoda H, Yokota-Ikeda N, Oshikawa S, Kanno Y, Yoshinaga K, Uchida K, et al. Decreased abundance of urinary exosomal aquaporin-1 in renal ischemia-reperfusion injury. Am J Physiol Renal Physiol. 2009;297(4):F1006-16.

20. Abdeen A, Sonoda H, El-Shawarby R, Takahashi S, Ikeda M. Urinary excretion pattern of exosomal aquaporin-2 in rats that received gentamicin. Am J Physiol Renal Physiol. 2014;307(11):F1227-37.

21. Sonoda H, Oshikawa-Hori S, Ikeda M. An Early Decrease in Release of Aquaporin-2 in Urinary Extracellular Vesicles After Cisplatin Treatment in Rats. Cells. 2019;8(2).

22. Oshikawa-Hori S, Yokota-Ikeda N, Sonoda H, Ikeda M. Urinary extracellular vesicular release of aquaporins in patients with renal transplantation. BMC nephrology. 2019;20(1):216.

23. Rossi L, Nicoletti MC, Carmosino M, Mastrofrancesco L, Di Franco A, Indrio F, et al. Urinary Excretion of Kidney Aquaporins as Possible Diagnostic Biomarker of Diabetic Nephropathy. Journal of diabetes research. 2017;2017:4360357.

24. Braun F, Rinschen M, Buchner D, Bohl K, Plagmann I, Bachurski D, et al. The proteomic landscape of small urinary extracellular vesicles during kidney transplantation. J Extracell Vesicles. 2020;10(1):e12026.

25. El Fekih R, Hurley J, Tadigotla V, Alghamdi A, Srivastava A, Coticchia C, et al. Discovery and Validation of a Urinary Exosome mRNA Signature for the Diagnosis of Human Kidney Transplant Rejection. J Am Soc Nephrol. 2021.
