# Supplementary material for: Fasting before living-kidney donation: effect on donor well-being and postoperative recovery: study protocol of a multicenter randomized controlled trial
Source: Trials. 2022 Jan 6;23:18. doi: 10.1186/s13063-021-05950-x (PMC8733810; doi:10.1186/s13063-021-05950-x)
Supplement: Supplementary file 2 — Additional file 2:. Informed consent forms. [file 13063_2021_5950_MOESM2_ESM.docx]

**Proefpersoneninformatie voor deelname
aan medisch-wetenschappelijk onderzoek**

**Effect van preoperatief vasten op herstel na nierdonatie**

*Officiële titel:* *Preoperatief vasten en herstel na nierdonatie, effect op postoperatieve vermoeidheid.*

**Inleiding**

Geachte heer/mevrouw,

Met deze informatiebrief willen we u vragen of u wilt meedoen aan medisch-wetenschappelijk onderzoek. Meedoen is vrijwillig. U krijgt deze brief omdat u heeft besloten een nier te gaan doneren en hiervoor binnenkort een ingreep ondergaat. U leest hier om wat voor onderzoek het gaat, wat het voor u betekent en wat de voordelen en nadelen zijn. Het is veel informatie. Wilt u de informatie doorlezen en beslissen of u wilt meedoen? Als u wilt meedoen, kunt u het formulier invullen dat u vindt in **bijlages E & F**.

**Stel uw vragen**

U kunt uw beslissing nemen met de informatie die u in deze informatiebrief vindt. Daarnaast raden we u aan om dit te doen:

- Stel vragen aan de onderzoeker die u deze informatie geeft.
- Praat met uw partner, familie of vrienden over dit onderzoek.
- Stel vragen aan de onafhankelijk deskundige, dr. J. de Jonge.
- Lees de informatie op [www.rijksoverheid.nl/mensenonderzoek](http://www.rijksoverheid.nl/mensenonderzoek).

1. **Algemene informatie**

Dit onderzoek is opgezet door het Erasmus Universitair Medisch Centrum en wordt verricht door artsen in het Erasmus Universitair Medisch Centrum en het Universitair Medisch Centrum Groningen. Voor dit onderzoek zijn 180 proefpersonen uit Nederland nodig. De medisch-ethische toetsingscommissie Erasmus MC heeft dit onderzoek goedgekeurd.

**2. Wat is het doel van het onderzoek?**

Het doel van dit onderzoek is uitzoeken of vasten voor een operatie zorgt voor beter herstel na een operatie. Het vasten houdt in dat er tweeënhalve dag voor de operatie zo goed als niet gegeten mag worden. We willen kijken of een korte periode van minder eten het lichaam in een “beschermstand” zet en daardoor zorgt voor een betere weerstand tegen stress, die met een operatie samenhangt.

Door een operatie kan lichamelijke stress ontstaan, dus een dieet zou de uitkomst van de operatie en het herstel na de operatie kunnen verbeteren. Om dit te onderzoeken worden personen gevraagd die een nier doneren omdat zij gezond zijn.

1. **Wat is de achtergrond van het onderzoek?**

Verschillende onderzoeken hebben laten zien dat dieren langer leven als er minder calorieën in de voeding zitten dan normaal. Dit komt gedeeltelijk door een betere weerstand tegen oxidatieve stress. Oxidatieve stress ontstaat in situaties die schadelijk zijn voor het lichaam zoals warmte, UV-licht en roken. Oxidatieve stress is ook aanwezig tijdens een operatie. Eerder onderzoek met donoren die een nier hebben afgestaan en patiënten die een maagverkleining hebben ondergaan liet zien dat een dieet voor de operatie goed werd verdragen zonder een hoger risico op complicaties na de operatie. Ook werd er een sneller herstel van de nierfunctie bij zowel de donor als de ontvanger van de nier gezien. Dit willen we nu in een grotere groep mensen opnieuw onderzoeken en bevestigen.

1. **Hoe verloopt het onderzoek?**

U heeft een weloverwogen beslissing genomen om een nier te gaan doneren. Voorafgaand aan de operatie zult u onderzoeken doorlopen die standaard worden gedaan voor deze operatie. Dit onderzoek vindt parallel aan de reguliere behandeling voor nierdonatie en transplantatie plaats.

*Hoelang duurt het onderzoek?*

Doet u mee met het onderzoek? Het onderzoek duurt vanaf start deelname tot 3 maanden na de nierdonatie.

*Stap 1: bent u geschikt om mee te doen?*

We willen eerst weten of u geschikt bent om mee te doen. Wanneer u toestemming geeft en meedoet aan dit onderzoek controleert de onderzoeker de volgende gegevens voordat u mee kan doen:

- Uw medische voorgeschiedenis
- Uw gewicht
- Of u goedgekeurd bent voor nierdonatie

*Stap 2: de dieetbehandeling voor de operatie*

Voor dit onderzoek maken we 2 groepen, willekeurige loting bepaalt in welke groep u komt;

- Groep 1: U blijft gewoon eten zoals u gewend bent voor de operatie.
- Groep 2: U krijgt het dieet dat wij willen onderzoeken. Dit dieet bestaat uit vasten gedurende 2,5 dag voor de operatie. Vasten betekent dat u geen voedsel mag eten dat calorieën bevat. Het enige wat u mag eten is in totaal één portie snoepgroenten (zgn. snoeptomaatjes, komkommers of worteltjes). Daarnaast moet u wel voldoende drinken, maar dit mag enkel water of koffie/thee zonder melk of suiker zijn. Daarnaast mag u 4x per dag een kopje bouillon (Maggi ‘Opkikker’ 8 kcal per 160

ml). Het dieet gaat door tot de operatie. Na de operatie mag u eten zoveel u wilt omdat uit onderzoek is gebleken dat dan het vasten zijn beschermende werking heeft verricht. U zal naast het vasten voor de operatie ook 3 dagen voor de operatie starten met 1 keer per dag een zakje laxeermiddel. Dit zal een mild laxerend effect hebben, waardoor u minimaal 1x per dag ontlasting heeft.

*Stap 3: onderzoeken en metingen*

Voor het onderzoek is het nodig dat u 1 extra keer in de studielooptijd naar het ziekenhuis komt. Alle andere studiehandelingen vinden thuis plaats of op momenten dat u reeds in het ziekenhuis bent. Het extra bezoek duurt ongeveer 15 minuten.

We doen de volgende extra studie-gerelateerde onderzoeken:

- Eén week voor de operatie krijgt u een Smartwatch die u 24/7 dient te dragen. Deze Smartwatch houdt automatisch bij hoeveel u beweegt, zowel voor als na de operatie. Deze gegevens worden digitaal naar het Erasmus MC gestuurd. De Smartwatch mag 2 weken na de operatie weer afgedaan en ingeleverd worden.
- Tijdens de operatie worden twee hapjes (zogenaamde biopten) genomen uit de nier: één hapje vlak na uitname van de nier en één hapje vlak na transplantatie in de ontvanger. De hapjes geven geen tot zeer weinig extra risico. Er bestaat een zeer kleine kans (+/- 1%) op een bloeding na het nemen van het biopt. Deze bloeding kan snel tijdens de operatie worden verholpen, waardoor de ontvanger er geen last van zal hebben. Ook wordt er een klein stukje van de afvoerbuis van de nier afgenomen voor weefselonderzoek. Dit stukje weefsel is niet nodig voor de transplantatie. De afvoerbuis van de nier is namelijk te lang voor de nieuwe plek in de ontvanger. Daarnaast wordt er een stukje resterend vetweefsel bewaard voor onderzoek, dit vetweefsel wordt normaliter tijdens de operatie verwijderd.
- Vaak produceert de nier ook direct urine tijdens de operatie. Is dit het geval, dan bewaren we deze urine. Naast het nemen van de biopten en het bewaren van de eerste urine zal de operatie gaan zoals dat standaard gaat. Na de operatie worden de stukjes weefsel door de onderzoekers onder de microscoop bekeken en onderzocht om te kijken of er een verschil is tussen de 2 studiegroepen.
- Alles bij elkaar nemen we 20ml extra bloed bij u af. Deze hoeveelheid geeft bij volwassenen geen problemen. Ter vergelijking: iemand die bloed geeft bij de bloedbank, geeft per keer 500ml bloed. Met het bloedonderzoek testen we de nierfunctie 4 weken na de operatie.
- Wij sturen u 5 keer twee korte vragenlijsten. De vragen gaan over vermoeidheid, uw algehele gezondheid en uw kwaliteit van leven. Het kost u ongeveer 10 minuten om deze vragenlijsten in te vullen. Deze vragenlijsten vult u 2x voor de operatie, tijdens opname en op 1 maand en 3 maanden na de operatie in.

De volgende onderzoeken gebeuren reeds i.v.m. standaardzorg, maar de resultaten hiervan zullen we meenemen in het onderzoek:

- Tijdens uw opname zal de prikzuster van de afdeling buisjes bloed afnemen. Dit gebeurt normaal gesproken op de volgende tijden: voor de operatie, 6 uur na de operatie en vervolgens elke ochtend totdat u met ontslag gaat. Naast de bloedwaardes die standaard worden bepaald bij een nierdonatie zullen er nog een aantal extra bepalingen worden gedaan. Tijdens opname wordt u dus niet vaker geprikt dan normaal.

Daarnaast is er per deelnemend centrum sprake van een extra studiehandeling. Zie hiervoor **Bijlage C: Centrum-specifiek onderzoek.**

Een overzicht van alle handelingen vindt u in **Bijlage D: Schema onderzoekshandelingen.**

*Stap 4: nacontrole*

Drie maanden na de nierdonatie vindt de laatste controle plaats. Op hetzelfde moment heeft u een afspraak bij de arts of verpleegkundig specialist die de verdere normale nacontroles i.v.m. nierdonatie met u zal bespreken.

*Wat is er anders dan bij gewone zorg?*

Veel studiehandelingen vinden parallel aan de normale behandeling rondom nierdonatie plaats. Kort samengevat is het volgende anders dan bij normale zorg:

- Het vasten voor de operatie, waarvoor u willekeurig wordt geloot.
- Het 3 weken dragen van een Smartwatch.
- Tijdens operatie worden 2 kleine weefselhapjes van de nier afgenomen en wordt er restweefsel afgenomen.
- Urine van voor, tijdens en na de operatie wordt onderzocht.
- Het 5 keer invullen van 2 korte vragenlijsten.
- Eén extra fysieke controle in het ziekenhuis, 4 weken na nierdonatie.

**Dossieronderzoek ontvanger**

Bij de ontvanger van de donornier wordt dossieronderzoek verricht. Er wordt gekeken naar de nierfunctie na de operatie en de mate van voorkomen van vertraagde functie of acute afstoting van de nier. In het kader van het onderzoek worden via het elektronische patiëntendossier deze gegevens verzameld.

1. **Welke afspraken maken we met u?**

We willen graag dat het onderzoek goed verloopt. Daarom maken we de volgende afspraken met u:

- U volgt het dieet dat u is voorgeschreven.
- U doet niet zonder medeweten van de onderzoeker ook nog aan een ander medisch-wetenschappelijk onderzoek mee.
- U komt naar iedere afspraak.
- U draagt het eventuele deelnemersboekje van het onderzoek bij u. Hierop staat dat u meedoet aan dit onderzoek en wie men moet waarschuwen bij een noodsituatie. Laat dit boekje zien als u bij een arts komt.
- U neemt contact op met de onderzoeker in deze situaties:
  - U wilt andere medicijnen gaan gebruiken. Ook als dit homeopathische middelen zijn, natuurgeneesmiddelen, vitaminen of geneesmiddelen van de drogist.
  - U wordt in een ziekenhuis opgenomen of behandeld.
  - U krijgt plotseling problemen met uw gezondheid.
  - U wilt niet meer meedoen met het onderzoek.
  - Uw telefoonnummer, adres of e-mailadres verandert.

*Mag u of uw partner zwanger worden tijdens het onderzoek?*

Vrouwen die zwanger zijn of borstvoeding geven, kunnen op dat moment niet hun nier doneren en zodoende niet meedoen aan dit onderzoek. Vrouwen mogen ook niet zwanger worden tijdens de voorbereiding voor nierdonatie en zodoende ook niet tijdens het onderzoek.

*Toch zwanger?*

Wordt u toch zwanger tijdens het onderzoek? Laat dit dan meteen weten aan de onderzoeker. U moet dan in overleg met de onderzoeker en uw behandelend arts mogelijk stoppen met dit onderzoek.

1. **Van welke bijwerkingen, nadelige effecten of ongemakken kunt u last krijgen?**

U kunt tijdens het dieet mogelijk een hongergevoel ervaren of licht draaierig worden. Is dit het geval, dan kunt u dit aangeven op de vragenlijsten en/of ten tijde van de opname aan de arts-onderzoeker/verpleegkundige. Daarnaast is het dieet een verandering in uw dagelijks leven en kan het zodoende gepaard gaan met stress. Het nemen van de biopten tijdens de operatie heeft geen tot zeer weinig extra risico.

1. **Wat zijn de voordelen en de nadelen als u meedoet aan het onderzoek?**

Meedoen aan het onderzoek kan voordelen en nadelen hebben. Hieronder zetten we ze op een rij. Denk hier goed over na en praat erover met anderen.

Het onderzoek kijkt naar de effecten van een dieet op het herstel na een operatie. Eerdere studies naar dit onderwerp lieten geen nadelige effecten, maar juist enkele voordelige effecten zien voor de donor of voor de ontvanger. We verwachten dat deze studie in de toekomst bijdraagt aan beter herstel na een operatie voor zowel de donor als de ontvanger.

Een mogelijk voordeel voor u als deelnemer is dat u minder vermoeid bent, uw nierfunctie sneller herstelt en de nierfunctie van de ontvanger sneller herstelt, maar zeker is dat niet.

Meedoen aan het onderzoek kan deze nadelen hebben:

- U kunt last krijgen van de bijwerkingen of nadelige effecten van het vastendieet zoals beschreven in paragraaf 6.
- U kunt last hebben van de metingen tijdens het onderzoek. Bijvoorbeeld: de bloedafname kan wat pijn doen. Of u kunt daardoor een bloeduitstorting krijgen.
- Meedoen aan het onderzoek kost u extra tijd:
  - Aan het invullen van vragenlijsten: 5 keer 10 minuten.
  - Eenmalig een extra afspraak in het ziekenhuis.
- Er bestaat een zeer kleine kans (+/-1%) op een bloeding na het weefselhapje uit de nier, dit kunnen we tijdens de operatie wel direct behandelen.
- U moet zich houden aan de afspraken die horen bij het onderzoek.

*Wilt u niet meedoen?*

U beslist zelf of u meedoet aan het onderzoek. Wilt u niet meedoen? Dan krijgt u de gewone behandeling voor de nierdonatie.

1. **Wanneer stopt het onderzoek?**

De onderzoeker laat het u weten als er nieuwe informatie over het onderzoek komt die belangrijk voor u is. De onderzoeker vraagt u daarna of u blijft meedoen.

In deze situaties stopt voor u het onderzoek:

- Alle onderzoeken volgens het schema zijn voorbij.
- U bent zwanger geworden.
- U wilt zelf stoppen met het onderzoek. Dat mag op ieder moment. Meld dit dan meteen bij de onderzoeker. U hoeft er niet bij te vertellen waarom u stopt. U krijgt dan weer de gewone behandeling voor de nierdonatie. De onderzoeker kan voor uw veiligheid nog een of meer controles afspreken.
- De onderzoeker vindt het beter voor u om te stoppen. De onderzoeker zal u nog wel uitnodigen voor een nacontrole.
- Een van de volgende instanties besluit dat het onderzoek moet stoppen:
  - Het Erasmus MC,
  - De overheid, of
  - De medisch-ethische commissie die het onderzoek beoordeelt.

*Wat gebeurt er als u stopt met het onderzoek?*

De onderzoekers gebruiken de gegevens en het lichaamsmateriaal (bloed, nier- en restweefsel) die tot het moment van stoppen zijn verzameld. Als u wilt, kan verzameld lichaamsmateriaal worden vernietigd. Geef dit door aan de onderzoeker.

Het hele onderzoek is afgelopen als alle deelnemers klaar zijn.

1. **Wat gebeurt er na het onderzoek?**

*Krijgt u de resultaten van het onderzoek?*

Ongeveer 2-3 jaar na uw deelname laat de onderzoeker u weten wat de belangrijkste uitkomsten zijn van het onderzoek, als u dit wenst. Wilt u dit niet weten? Zeg dat dan tegen de onderzoeker. Hij zal het u dan niet vertellen.

1. **Wat doen we met uw gegevens en lichaamsmateriaal?**

Doet u mee met het onderzoek? Dan geeft u ook toestemming om uw gegevens en lichaamsmateriaal te verzamelen, gebruiken en bewaren.

*Welke gegevens bewaren we?*

We bewaren de volgende gegevens:

Uw naam

Uw geslacht

Uw adres

Uw geboortedatum

Gegevens over uw gezondheid

(Medische) gegevens die we tijdens het onderzoek verzamelen

*Welk lichaamsmateriaal bewaren we?*

We bewaren de buisjes bloed, de urine en stukjes weefsel verkregen tijdens de operatie.

*Waarom verzamelen, gebruiken en bewaren we uw gegevens en lichaamsmateriaal?*

We verzamelen, gebruiken en bewaren uw gegevens en uw lichaamsmateriaal om de vragen van dit onderzoek te kunnen beantwoorden en om de resultaten te kunnen publiceren.

*Hoe beschermen we uw privacy?*

Om uw privacy te beschermen geven wij uw gegevens en uw lichaamsmateriaal een code. Op al uw gegevens en lichaamsmateriaal zetten we alleen deze code. De sleutel van de code bewaren we op een beveiligde plek in uw eigen ziekenhuis. Als we uw gegevens en

lichaamsmateriaal verwerken, gebruiken we steeds alleen die code. Ook in rapporten en publicaties over het onderzoek kan niemand terughalen dat het over u ging.

*Wie kunnen uw gegevens zien?*

Sommige personen kunnen wel uw naam en andere persoonlijke gegevens zonder code inzien. Dit zijn mensen die controleren of de onderzoekers het onderzoek goed en betrouwbaar uitvoeren. Deze personen kunnen bij uw gegevens komen:

- Leden van de commissie die de veiligheid van het onderzoek in de gaten houden.
- Een controleur die door het Erasmus Medisch Centrum is ingehuurd of een controleur die voor het Erasmus Medisch Centrum werkt.
- Nationale en internationale toezichthoudende autoriteiten. Bijvoorbeeld de Inspectie Gezondheidszorg en Jeugd.

Deze personen houden uw gegevens geheim. Wij vragen u voor deze inzage toestemming te geven.

*Hoelang bewaren we uw gegevens en lichaamsmateriaal?*

We bewaren uw gegevens 20 jaar op de onderzoekslocatie. Uw lichaamsmateriaal bewaren we ook op de onderzoekslocatie. Het wordt tijdens de studie, ongeveer 2-3 jaar, bewaard om daarop in de loop van dit onderzoek nog nieuwe bepalingen te kunnen doen die te maken hebben met dit onderzoek. Zodra dit niet meer nodig is, vernietigen we uw lichaamsmateriaal.

*Mogen we uw gegevens en lichaamsmateriaal gebruiken voor ander onderzoek?*

Uw gegevens en uw (overgebleven) lichaamsmateriaal kunnen na afloop van dit onderzoek ook nog van belang zijn voor ander wetenschappelijk onderzoek op het gebied van het vastendieet en operaties. Daarvoor zullen uw gegevens en lichaamsmateriaal 15 jaar worden bewaard op de onderzoekslocatie. In het toestemmingformulier geeft u aan of u dit goed vindt. Geeft u geen toestemming? Dan kunt u nog steeds meedoen met dit onderzoek. U krijgt dezelfde zorg.

*Wat gebeurt er bij onverwachte ontdekkingen?*

Tijdens het onderzoek kunnen we toevallig iets vinden dat belangrijk is voor uw gezondheid. De onderzoeker neemt dan contact op met uw huisarts en behandelend arts. U bespreekt dan met uw huisarts of specialist wat er moet gebeuren. U geeft met het formulier toestemming voor het informeren van uw huisarts of specialist.

*Kunt u uw toestemming voor het gebruik van uw gegevens weer intrekken?*

U kunt uw toestemming voor het gebruik van uw gegevens op ieder moment intrekken. Maar let op: trekt u uw toestemming in, en hebben onderzoekers dan al gegevens verzameld voor een onderzoek? Dan mogen zij deze gegevens nog wel gebruiken. Voor uw lichaamsmateriaal geldt dat de onderzoekers dit vernietigen nadat u uw toestemming intrekt. Maar zijn er dan al metingen gedaan met uw lichaamsmateriaal? Dan mag de onderzoeker de resultaten daarvan blijven gebruiken.

*Wilt u meer weten over uw privacy?*

- Wilt u meer weten over uw rechten bij de verwerking van persoonsgegevens? Kijk dan op [www.autoriteitpersoonsgegevens.nl](http://www.autoriteitpersoonsgegevens.nl).
- Heeft u vragen over uw rechten? Of heeft u een klacht over de verwerking van uw persoonsgegevens? Neem dan contact op met degene die verantwoordelijk is voor de verwerking van uw persoonsgegevens. Voor uw onderzoek is dat: het Erasmus Medisch Centrum, zie **bijlage A** voor de contactgegevens en de website.
- Als u klachten heeft over de verwerking van uw persoonsgegevens, raden we u aan om deze eerst te bespreken met het onderzoeksteam. U kunt ook naar de Functionaris Gegevensbescherming van het Erasmus Medisch Centrum gaan. Of u dient een klacht in bij de Autoriteit Persoonsgegevens.

*Waar vindt u meer informatie over het onderzoek?*

Op de volgende website vindt u meer informatie over het onderzoek: Nederlands Trial Register ([www.https://www.trialregister.nl/](http://www.https://www.trialregister.nl/)). Na het onderzoek kan de website een samenvatting van de resultaten van dit onderzoek tonen. U vindt het onderzoek door te zoeken op ‘FAST-Studie’.

1. **Krijgt u een vergoeding als u meedoet aan het onderzoek?**

De extra testen en behandeling voor het onderzoek kosten u niets. U krijgt ook geen vergoeding als u meedoet aan dit onderzoek.

1. **Bent u verzekerd tijdens het onderzoek?**

Voor iedereen die meedoet aan dit onderzoek is een verzekering afgesloten. De verzekering betaalt voor schade door het onderzoek. Maar niet voor alle schade. In **bijlage B** vindt u meer informatie over de verzekering en de uitzonderingen. Daar staat ook aan wie u schade kunt melden.

1. **We informeren uw huisarts en behandelend specialist**

De onderzoeker stuurt uw huisarts en behandelend specialist een brief om te laten weten dat u meedoet aan het onderzoek. Dit is voor uw eigen veiligheid.

1. **Heeft u vragen?**

Vragen over het onderzoek kunt u stellen aan de onderzoeker. Wilt u advies van iemand die er geen belang bij heeft? Ga dan naar de onafhankelijke arts dr. J. de Jonge. Hij weet veel over het onderzoek, maar werkt niet mee aan dit onderzoek.

Heeft u een klacht? Bespreek dit dan met de onderzoeker of de arts die u behandelt. Wilt u dit liever niet? Ga dan naar klachtenfunctionaris. In **bijlage A** staat waar u die kunt vinden.

1. **Hoe geeft u toestemming voor het onderzoek?**

U kunt eerst rustig nadenken over dit onderzoek. Daarna vertelt u de onderzoeker of u de informatie begrijpt en of u wel of niet wilt meedoen. Wilt u meedoen? Dan vult u het toestemmingsformulier in dat u bij deze informatiebrief vindt. U en de onderzoeker krijgen allebei een getekende versie van deze toestemmingsverklaring.

Dank voor uw tijd.

Met vriendelijke groet,

Prof. dr. J.N.M. IJzermans Dr. R.C. Minnee

Hoofd afdeling HPB & Transplantatie Transplantatiechirurg

Hepatobiliair- en Transplantatiechirurg *Erasmus MC*

*Erasmus MC*

Dr. R.A. Pol

Hoofd Transplantatie en Orgaandonatie

Transplantatiechirurg

*UMC Groningen*

Prof. Dr. J.H.J. Hoeijmakers Drs. C.A.J. Oudmaijer

Hoogleraar Moleculaire Genetica Arts-onderzoeker

*Erasmus MC* *Erasmus MC*

1. **Bijlagen bij deze informatie**

A. Contactgegevens

B. Informatie over de verzekering

C. Centrum-specifiek onderzoek

D. Schema onderzoekshandelingen

E. Toestemmingsformulier proefpersoon

F. Toestemmingsformulier ontvanger

**Bijlage A: Contactgegevens**

Hoofdonderzoeker:

Prof. Dr. J.N.M. IJzermans, Hoofd Transplantatie

Bereikbaar via telefoonnummer: 010-7031810.

Plaatselijke Hoofdonderzoeker:

Dr. R.C. Minnee, transplantatiechirurg

Bereikbaar via telefoonnummer: 010-7031810.

Coördinerend onderzoeker:

C.A.J. Oudmaijer, arts-onderzoeker

E-mail: [FAST.Studie@erasmusmc.nl](mailto:FAST.Studie@erasmusmc.nl)

Bereikbaar via telefoonnummer: 010-7043541 / 06-50173127.

Onafhankelijk deskundige

Dr. J. de Jonge, Hepato-Pancreato-Biliair en Transplantatiechirurg

Hij weet veel over het onderzoek, maar heeft niets te maken met dit onderzoek. U kunt hem in het Erasmus MC bereiken via telefoonnummer: 010-7031810.

Klachten:

Digitaal: via het digitale klachtenformulier. Dit wordt automatisch verzonden naar het mailadres [klachtenopvang@erasmusmc.nl](mailto:klachtenopvang@erasmusmc.nl).
Telefonisch: 010-7033198
Per post: Erasmus MC Klachtenopvang, Antwoordnummer 55, 3000 WB Rotterdam

Tevens kunt u uw klachten melden aan de onderzoeker of uw behandelend arts.

Functionaris voor de Gegevensbescherming van het Erasmus MC

De functionaris gegevensbescherming is te bereiken via het Secretariaat Juridische Zaken tijdens kantoortijden op tel. 010-7034986.

Voor meer informatie over uw rechten: <https://www.erasmusmc.nl/nl-nl/patientenzorg/privacy>

**Bijlage B: informatie over de verzekering**

Het Erasmus MC heeft een verzekering afgesloten voor iedereen die meedoet aan het onderzoek. De verzekering betaalt de schade die u heeft doordat u aan het onderzoek meedeed. Het gaat om schade die u krijgt tijdens het onderzoek, of binnen 4 jaar na het onderzoek. U moet schade binnen 4 jaar melden bij de verzekeraar.

Heeft u schade door het onderzoek? Meld dit dan bij deze verzekeraar:

De verzekeraar van het onderzoek is:

Naam: CNA Insurance Company (Europe) S.A.

Adres: Polarisavenue 140, 2134 JX Hoofddorp

Telefoonnummer: + 31 (0)23-3036004, + 31 (0)6 380 59413

E-mail: ClaimsNetherlands@cnahardy.com of esther.vanherk@cnaeurope.com

Contactpersoon: Esther van Herk, Senior Claims Handler

De verzekering betaalt maximaal € 650.000 per persoon en een maximumbedrag van € 5.000.000 voor het hele onderzoek en maximaal € 7.500.000 per jaar voor alle onderzoeken van dezelfde opdrachtgever.

Let op: de verzekering dekt de volgende schade **niet**:

- Schade door een risico waarover we u informatie hebben gegeven in deze brief. Maar dit geldt niet als het risico groter bleek te zijn dan we van tevoren dachten. Of als het risico heel onwaarschijnlijk was.
- Schade aan uw gezondheid die ook zou zijn ontstaan als u niet aan het onderzoek had meegedaan.
- Schade die ontstaat doordat u aanwijzingen of instructies niet of niet goed opvolgde.
- Schade aan de gezondheid van uw kinderen of kleinkinderen.
- Schade door een behandelmethode die al bestaat. Of door onderzoek naar een behandelmethode die al bestaat.

Deze bepalingen staan in het 'Besluit verplichte verzekering bij medisch-wetenschappelijk onderzoek met mensen 2015'. Dit besluit staat in de Wettenbank van de overheid (<https://wetten.overheid.nl>).

**Bijlage C: Centrum-specifiek onderzoek**

In elk deelnemend centrum is er sprake van een klein aanvullend medisch-wetenschappelijk onderzoek, dit ter aanvulling op onze basis-onderzoeksvraag. Hieronder vindt u voor het ziekenhuis waar u meedoet aan de studie wat de aanvullende studiehandelingen zijn.

Erasmus Medisch Centrum

Binnen het Erasmus Medisch Centrum vindt een aanvullend onderzoek plaats naar markers in de urine. Deze markers zouden een goede voorspeller kunnen zijn voor toekomstige nierschade en dus de uitkomst van de niertransplantatie. Dit onderzoeken we op elke studie locatie in de urine van tijdens de operatie, maar in het Erasmus MC zal er extra urine verzameld worden voor en na de operatie, namelijk als volgt;

**Donor:** U levert eenmalig urine voor de operatie en eenmalig urine na de donatie in voor aanvullend onderzoek.

**Ontvanger:** U levert eenmalig urine voor de operatie en eenmalig urine na de operatie in voor aanvullend onderzoek.

**Bijlage D: Schema onderzoekshandelingen**

### Bijlage D.1: Onderzoekshandelingen Donor

| **Bezoek** | **Wanneer?** | **Welke studiehandelingen?** |
| --- | --- | --- |
| T= -4 | Afspraak op polikliniek transplantiecoördinator | - De transplantiecoördinator kijkt of u geschikt bent om deel te nemen aan dit onderzoek. - Indien dit het geval is, krijgt u informatie van de studie en zal de transplantiecoördinator dit toelichten. - Indien u geïnteresseerd bent, kan een aanvullend gesprek plaatsvinden met de arts-onderzoeker. U ontvangt mondelinge en schriftelijke informatie. U krijgt gelegenheid om vragen te stellen. - U krijgt bedenktijd tot aan uw afspraak op de polikliniek chirurgie. |
| T= -3 | Afspraak op polikliniek transplantatiechirurgie | - Poliklinische afspraak met uw behandelend chirurg met informatie t.a.v. de normale zorg voor nierdonatie. - Voorafgaand aan de nierdonatie zult u onderzoeken doorlopen/ondergaan die standaard worden gedaan voor deze operatie. - Poliklinische afspraak met arts-onderzoeker t.a.v. studievoorlichting, beantwoorden van uw resterende vragen, gezamenlijk ondertekenen van toestemmingsformulier. - U wordt in een studiegroep geloot en krijgt uitgebreide voorlichting t.a.v. uw studiebehandeling. - U vult een tweetal vragenlijsten in, dit kan zowel digitaal als op papier. |
| T= -2 | 1 week voor de operatie | - U ontvangt de Smartwatch thuis met instructies en gaat deze 24 uur per dag dragen voor de komende 3 weken. |
| T= -1 | 3 dagen voor de operatie | - Indien u in de vastengroep zit, begint u 60 uur voor de operatie met vasten. Op diezelfde dag begint u met het nemen van het laxans, dit doet u 3x, 1x per dag tot de operatie. - U vult een tweetal vragenlijsten in, dit kan zowel digitaal als op papier. |
| T= 0 | Dag van de operatie | - Tijdens de operatie worden er een tweetal nierbiopten (weefselhapjes) afgenomen en wordt er restweefsel afgenomen voor onderzoek. - De urine die tijdens de operatie reeds door de nier wordt gemaakt, wordt opgestuurd voor onderzoek. - U levert ‘s ochtends en ‘s avond een beetje urine in voor onderzoek. |
| T= +1 | 1 dag na de operatie | - U wordt volgens de standaardzorg behandeld in het ziekenhuis. |
| T= +2 | 2 dagen na de operatie | - U wordt volgens de standaardzorg behandeld in het ziekenhuis. |
| T= +3 | 3 dagen na de operatie | - U wordt volgens de standaardzorg behandeld in het ziekenhuis. - Afhankelijk van hoe het gaat, gaat u vandaag met ontslag. Voor ontslag vult u een tweetal vragenlijsten in, dit kan zowel digitaal als op papier. |
| T= +4 | 4 dagen na de operatie | - U wordt volgens de standaardzorg behandeld in het ziekenhuis - Voor ontslag vult u een tweetal vragenlijsten in, dit kan zowel digitaal als op papier. |
| T= +5 | 2 weken na de operatie | - U doet de Smartwatch af en stuurt deze terug naar het Erasmus MC. |
| T= +6 | 4 weken na de operatie | - U komt voor een extra controle met bloedonderzoek naar het ziekenhuis. - U vult een tweetal vragenlijsten in, dit kan zowel digitaal als op papier. |
| T= +7 | 12 weken na de operatie | - U komt voor een reguliere controle met bloedonderzoek naar het ziekenhuis. U komt zowel bij de arts-onderzoeker voor het einde van de studie, als bij de verpleegkundig specialist die het verdere vervolgtraject met u bespreekt. - U vult een tweetal vragenlijsten in, dit kan zowel digitaal als op papier. |

### Bijlage D.2: Onderzoekshandelingen Ontvanger

| **Bezoek** | **Wanneer?** | **Welke studiehandelingen?** |
| --- | --- | --- |
| T= -2 | Afspraak op polikliniek transplantiecoördinator | - De transplantiecoördinator kijkt of u geschikt bent om deel te nemen aan dit onderzoek. - Indien dit het geval is, krijgt u informatie over de studie en zal de transplantiecoördinator dit toelichten. - Indien u geïnteresseerd bent, kan een aanvullend gesprek plaatsvinden met de arts-onderzoeker. U ontvangt mondelinge en schriftelijke informatie. U krijgt gelegenheid om vragen te stellen. - U krijgt bedenktijd tot aan uw afspraak op de polikliniek chirurgie. |
| T= -1 | Afspraak op polikliniek transplantatiechirurgie | - Poliklinische afspraak met uw behandelend chirurg met informatie t.a.v. de normale zorg voor niertransplantatie. - Voorafgaand aan de niertransplantatie zult u onderzoeken doorlopen/ondergaan die standaard worden gedaan voor deze operatie. - Poliklinische afspraak met arts-onderzoeker t.a.v. studievoorlichting, beantwoorden van uw resterende vragen, gezamenlijk ondertekenen van toestemmingsformulier. |
| T= 0 | Dag van de operatie | - Tijdens de operatie worden er een tweetal nierbiopten (weefselhapjes) afgenomen en wordt er restweefsel afgenomen voor onderzoek. - De urine die tijdens de operatie reeds door de nier wordt gemaakt, wordt opgestuurd voor onderzoek. - U levert ‘s ochtends en ‘s avond een beetje urine in voor onderzoek. |
| T= +1 | Na de operatie | - De arts-onderzoeker haalt gegevens uit het elektronisch patiëntendossier t.a.v. uw nierfunctie na de operatie en het herstel na de operatie. |

**Bijlage E: toestemmingsformulier proefpersoon (Donor)**

Behorende bij *Preoperatief vasten en herstel na nierdonatie, effect op postoperatieve vermoeidheid.*

- Ik heb de informatiebrief gelezen. Ook kon ik vragen stellen. Mijn vragen zijn goed genoeg beantwoord. Ik had genoeg tijd om te beslissen of ik meedoe.
- Ik weet dat meedoen vrijwillig is. Ook weet ik dat ik op ieder moment kan beslissen om toch niet mee te doen met het onderzoek. Of om ermee te stoppen. Ik hoef dan niet te zeggen waarom ik wil stoppen.
- Ik geef de onderzoeker toestemming om mijn huisarts/specialist(en) die mij behandelt te laten weten dat ik meedoe aan dit onderzoek.
- Ik geef de onderzoeker toestemming om informatie op te vragen bij mijn huisarts/specialist(en) die mij behandelt over mijn medische voorgeschiedenis.
- Ik geef de onderzoeker toestemming om mijn huisarts of specialist informatie te geven over onverwachte bevindingen uit het onderzoek die van belang zijn voor mijn gezondheid.
- Ik geef de onderzoekers toestemming om mijn gegevens en lichaamsmateriaal te verzamelen en gebruiken. De onderzoekers doen dit alleen om de onderzoeksvraag van dit onderzoek te beantwoorden.
- Ik weet dat voor de controle van het onderzoek sommige mensen al mijn gegevens kunnen inzien. Die mensen staan in deze informatiebrief. Ik geef deze mensen toestemming om mijn gegevens in te zien voor deze controle.
- Wilt u in de tabel hieronder ja of nee aankruisen?

| Ik geef toestemming om mijn gegevens te bewaren om dit te gebruiken voor ander onderzoek, zoals in de informatiebrief staat. | Ja ☐ | Nee☐ |
| --- | --- | --- |
| Ik geef toestemming om mijn (overgebleven) lichaamsmateriaal te bewaren om dit te gebruiken voor ander onderzoek, zoals in de informatiebrief staat. Het lichaamsmateriaal wordt daarvoor nog 15 jaar bewaard. | Ja ☐ | Nee☐ |
| Ik geef toestemming om mij eventueel na dit onderzoek te vragen of ik wil meedoen met een vervolgonderzoek. | Ja ☐ | Nee☐ |

- Ik wil meedoen aan dit onderzoek.

Mijn naam is (proefpersoon): ………………………………..

Handtekening: ……………………… Datum : __ / __ / __

-----------------------------------------------------------------------------------------------------------------

Ik verklaar dat ik deze proefpersoon volledig heb geïnformeerd over het genoemde onderzoek.

Wordt er tijdens het onderzoek informatie bekend die de toestemming van de proefpersoon kan beïnvloeden? Dan laat ik dit op tijd weten aan deze proefpersoon.

Naam onderzoeker (of diens vertegenwoordiger):……………………………….

Handtekening:……………………… Datum: __ / __ / __

-----------------------------------------------------------------------------------------------------------------

Aanvullende informatie is gegeven door:

Naam:………………………………..

Functie:………………………………

Handtekening:……………………… Datum: __ / __ / __

-----------------------------------------------------------------------------------------------------------------

*De proefpersoon krijgt een volledige informatiebrief mee, samen met een getekende versie van het toestemmingsformulier.*

**Bijlage F: toestemmingsformulier onderzoek (Ontvanger)**

Behorende bij *Preoperatief vasten en herstel na nierdonatie, effect op postoperatieve vermoeidheid.*

- Ik heb de informatiebrief gelezen. Ook kon ik vragen stellen. Mijn vragen zijn goed genoeg beantwoord. Ik had genoeg tijd om te beslissen of ik meedoe.
- Ik weet dat meedoen vrijwillig is. Ook weet ik dat ik op ieder moment kan beslissen om toch niet mee te doen met het onderzoek. Of om ermee te stoppen. Ik hoef dan niet te zeggen waarom ik wil stoppen.
- Ik geef de onderzoeker toestemming om mijn huisarts/specialist(en) die mij behandelt te laten weten dat ik meedoe aan dit onderzoek.
- Ik geef de onderzoeker toestemming om informatie op te vragen bij mijn huisarts/specialist(en) die mij behandelt over mijn medische voorgeschiedenis.
- Ik geef de onderzoeker toestemming om mijn huisarts of specialist informatie te geven over onverwachte bevindingen uit het onderzoek die van belang zijn voor mijn gezondheid.
- Ik geef de onderzoekers toestemming om mijn gegevens en lichaamsmateriaal te verzamelen en gebruiken. De onderzoekers doen dit alleen om de onderzoeksvraag van dit onderzoek te beantwoorden.
- Ik weet dat voor de controle van het onderzoek sommige mensen al mijn gegevens kunnen inzien. Die mensen staan in deze informatiebrief. Ik geef deze mensen toestemming om mijn gegevens in te zien voor deze controle.
- Wilt u in de tabel hieronder ja of nee aankruisen?

| Ik geef toestemming om mijn gegevens te bewaren om dit te gebruiken voor ander onderzoek, zoals in de informatiebrief staat. | Ja ☐ | Nee☐ |
| --- | --- | --- |
| Ik geef toestemming om mijn (overgebleven) lichaamsmateriaal te bewaren om dit te gebruiken voor ander onderzoek, zoals in de informatiebrief staat. Het lichaamsmateriaal wordt daarvoor nog 15 jaar bewaard. | Ja ☐ | Nee☐ |
| Ik geef toestemming om mij eventueel na dit onderzoek te vragen of ik wil meedoen met een vervolgonderzoek. | Ja ☐ | Nee☐ |

- Ik wil meedoen aan dit onderzoek.

Mijn naam is (proefpersoon): ………………………………..

Handtekening: ……………………… Datum : __ / __ / __

-----------------------------------------------------------------------------------------------------------------

Ik verklaar dat ik deze proefpersoon volledig heb geïnformeerd over het genoemde onderzoek.

Wordt er tijdens het onderzoek informatie bekend die de toestemming van de proefpersoon kan beïnvloeden? Dan laat ik dit op tijd weten aan deze proefpersoon.

Naam onderzoeker (of diens vertegenwoordiger):……………………………….

Handtekening:……………………… Datum: __ / __ / __

-----------------------------------------------------------------------------------------------------------------

Aanvullende informatie is gegeven door:

Naam:………………………………..

Functie:………………………………

Handtekening:……………………… Datum: __ / __ / __

-----------------------------------------------------------------------------------------------------------------

*De proefpersoon krijgt een volledige informatiebrief mee, samen met een getekende versie van het toestemmingsformulier.*
