# Supplementary material for: Fasting before living-kidney donation: effect on donor well-being and postoperative recovery: study protocol of a multicenter randomized controlled trial
Source: Trials. 2022 Jan 6;23:18. doi: 10.1186/s13063-021-05950-x (PMC8733810; doi:10.1186/s13063-021-05950-x)
Supplement: Supplementary file 3 — Additional file 3:. Rebuttal letter. [file 13063_2021_5950_MOESM3_ESM.pdf]

Editors-in-Chief, Trials
Prof. Sunil Badve, Associate Editor
The George Institute for Global Health, Australia

Concerning: (TRLS-D-21-00898) titled "Fasting before living-kidney donation: effect on donor wellbeing and postoperative recovery: Study protocol of a multicentre randomised controlled trial"

15-12-2021, Rotterdam, the Netherlands

Dear prof. Sunil Badve,

Thank you for giving us the opportunity to respond to the comments of the reviewers on the manuscript entitled "Fasting before living-kidney donation: effect on donor wellbeing and postoperative recovery".

We want to thank the reviewers for their time and helpful suggestions and have accordingly made several adjustments that raised the quality of our manuscript. Please find our point-by-point answers indicating the changes we have made below. Besides a clean manuscript version, we have also uploaded one highlighting all changes.

Reviewer reports:
Reviewer #1: Thank you for the opportunity to review the manuscript (TRLS-D-21-00898) titled "Fasting before living-kidney donation: effect on donor wellbeing and postoperative recovery: Study protocol of a multicentre randomised controlled trial". Please see my comments below:

1) The biological benefits of caloric restriction such as immunomodulation, anti-oxidative effects and reduced ischaemia-reperfusion are predominantly based on experimental or animal studies with much less data in humans. This needs to be highlighted more strongly in the background text (page 6, lines 90-91 and lines 101-102). Currently this reads as if this is already well established in human subjects as opposed to requiring translational-type research to confirm basic science observations.

> *Thank you for your comment. We have adjusted this in the new version of the manuscript, better reflecting that this is predominantly based on experimental and/or animal studies (Manuscript – Tracked Changes (All Markup), page 6, section 2, lines 82 and 88).*

2) The authors have discussed in fairly large detail (in the background section) the underlying biological mechanisms of how caloric restriction may improve ischaemia reperfusion injury, acute

stress responses, anti-ageing and the potential for reducing delayed graft function through immunodulation. However, there has been no discussion specifically linking these pathways with fatigue. Exactly how do these pathways cause or relate to fatigue? In other words, why was fatigue chosen as the primary endpoint for this study?

Thank you for your comment. We have adjusted this in the new version of the manuscript, providing more clear explanation why we think short-term fasting influences and could reduce fatigue (Manuscript – Tracked Changes (All Markup), page 7, section 2, lines 123-125). We hypothesize that by reducing caloric intake through STF the systemic inflammatory response may be attenuated and thus, postoperative fatigue, especially notable during the first three months in living-kidney donors, might be diminished.

3) The RAND-36 examines a number of domains which include both physical and mental health parameters. Which of these will be used to specifically assess fatigue or is it simply the aggregate score used to judge the endpoint? Also, does a higher aggregate score signify better functionality/QOL or vice versa? Some of the domains such as 'happiness' are marked that a higher 'score' is associated with being happy 'none of the time'. There should be some brief discussion as to the significance of the RAND-36 score (ie. high or low) and which measures are relevant to fatigue

We will use the domains "Physical functioning", "Energy/fatigue" and the aggregate score. We have provided more information regarding significance of the RAND-36 score with appropriate referencing in the new version of the manuscript (Manuscript – Tracked Changes (All Markup), page 9, section 4, lines 199-205). Absolute difference in the RAND-36 score ("Physical functioning", "Energy/fatigue" and the aggregate score) will be compared between both treatment arms. Reference data on postoperative fatigue after living-kidney donation is available from Klop et al (Transplantation Proceedings, 2018, PMID: 29407329). Literature on the RAND-36 shows that small differences could be interpreted as clinically important (Hays et al, Annals of medicine, 2001, PMID: 1149119).

4) How and why was the time frame of 60-hours pre-transplant surgery chosen? Is this based on previous clinical work or extrapolated from animal models demonstrating significant changes over this period?

Thank you for this critical question. Indeed, the time frame may be pivotal to find an effect. We selected the timing based on both previous clinical work and animal models. The 60-hour fasting diet was extrapolated from the animal models referenced in the manuscript (Jongbloed et al, PLoS One, 2014, PMID: 24959849 and/or Mitchell et al, Aging Cell, 2010, PMID: 19878145), while the earlier smaller clinical trials (van Ginhoven et al, Clinical Transplantation, 2011, PMID: 20718826, Jongbloed et al, Nutrients, 2016, PMID: 27213441, Jongbloed et al, Aging, 2020, PMID: 32652516, and/or de Groot et al, Nature Communications, 2020, PMID: 32576828) provided us with information on adherence and practical implications: this showed that long-term caloric restriction was more difficult to adhere to than short-term fasting. We have added this, for clarification, to the revised manuscript (Manuscript – Tracked Changes (All Markup), page 8, section 2, lines 152-154).

.

5) Is there going to be any monitoring of adherence to the dietary restriction beyond self-reported patient measures and change in body weight? Also, what measures would be taken to ensure that the control arm does not try and self-adopt a calorie restricted diet?

Thank you for this very relevant comment. Adherence to the protocol will be of critical importance to the outcome of our study. Adherence will be monitored by analysis of blood samples collected at the time of surgery. We will perform extensive analyses on these samples, e.g., investigating and comparing blood glucose levels in both treatment arms. Other important parameters are fasting insulin, albumin, urea and total cholesterol. If non-adherence in any of the two groups has happened but was not reported by the study subject, we expect to measure this in these blood samples, as stated in the manuscript (Manuscript – Tracked Changes (All Markup), page 10, section 1, lines 213-219).

We will inform all enrolled patients of the importance of a reliable control and intervention arm and therefore the importance of adhering to their assigned diet. The informed consent also clearly states that participants must be adherent tot the protocol or may withdraw otherwise. We will also provide a preoperative drink to the control-arm, current standard of care, before surgery, thereby making sure that they have caloric intake.

Finally, the dietary advice will be given by a specific dietitian or is this general advice as per the study coordinators?

Dietary advice is provided per the study coordinator, both orally and in writing, on two occasions (Manuscript – Tracked Changes (All Markup), page 8, section 2, lines 160-162).

6) The comparison of the primary endpoint is not entirely clear. Is the absolute difference in RAND-36 scores at 3 months going to be compared between the groups? Or is the percentage change from baseline from baseline pre-operative fatigue going to be compared?

Thank you for your valuable questions. We have further clarified this in the manuscript as indicated (Manuscript – Tracked Changes (All Markup), page 9, section 4, lines 199-205). The primary endpoint is the absolute difference in RAND-36 scores between both treatment arms at 4 weeks after surgery, since we expect baseline to be comparable due to adequate randomization. We have reference data on pre- and postoperative scores from the cohort from Klop et al (Transplantation Proceedings, 2018, PMID: 29407329). As secondary endpoint, we analyse the relative change in RAND-36 scores from baseline at several timepoints (3 days, 4 weeks and 3 months), using a multivariable linear regression model, mentioned in the section statistical analysis.

The authors have stated in the sample size calculation that '15% improvement in post-operative fatigue with a power of 0.95 as measured by the RAND-36'. Furthermore, is a 15% difference or change in RAND-36 considered clinically relevant?

Thank you for your comment. Indeed, we state that a 15% difference may be of clinical importance. We have included a reference on clinical relevance of change in scores in the RAND-36 (Hays et al, PMID: 11491194) and changed it in the manuscript (Manuscript – Tracked Changes (All Markup), page 9, section 4, lines 199-205).

As the reviewer suggests, it is generally believed that small differences in health related QoL are not important, even if statistical significance is achieved. Various papers have tried to answer the question on findings indicating a clinically relevant difference. They concluded that a small difference of 3-5 points in RAND translates into a 0.09-0.28 effect size. However, a critical evaluation is essential in which the patient cohort must also be considered. Living kidney donors may have a different frame of reference than patients and will therefore respond differently. Based on the literature and this consideration, we assume that a difference of 15% will certainly be clinically relevant for this specific healthy population.

7) Further leading from above, the authors also state in the statistical analysis section (page 12; line 290-291) that 'The expected decrease in post-operative fatigue due to the intervention will be assessed using a Wald test'. Exactly what is the threshold value for expected change (15%) and has this figure already been established in the literature?

We apologise for the unclear phrasing: this phrase meant to state that statistical significance will be assessed via the Wald test. It has been clarified in the manuscript (Manuscript – Tracked Changes (All Markup), page 14, section 2, line 306) and more information has been provided (Manuscript – Tracked Changes (All Markup), page 9, section 4, lines 199-205).

8) Would it be important to adjust for differences in baseline dietary habits between the groups?

The reviewer makes a fair comment, but given the randomization process we expect that any difference in baseline dietary habits between the two study groups will be non-existent.

9) HLA-incompatible transplantation is listed as an exclusion criteria. Does this imply that all transplants will be across only HLA-identical donor-recipient pairs? If so, what is the rationale for this?

Donor-recipient pairs do not need to be HLA-identical. HLA-incompatible implies that donor-recipient pairs with a positive crossmatch who participate in HLA-incompatible transplantation (with extensive pre-transplantation treatment/immunosuppression), are excluded from participation. We have clarified this in the manuscript (Manuscript – Tracked Changes (All Markup), page 8, section 1, line 148). Thank you for your comment.

Reviewer #2: Thanks for this interesting and well written protocol. I have a few comments based on the SPIRIT checklist. Numbers below refer to items on the checklist.

5b, c and d. Please complete these items in the checklist, they mustn't be left marked N/A without an explanation, and you have provided the information for 5b and 5c in the manuscript anyway.

Thank you for your comment, it has been completed in the revised version (Completed Spirit Checklist – Tracked Changes (All Markup), page 2, Section 2, 3 and 4).

11b can you give more detail on criteria for discontinuing or modifying the intervention

Thank you for your comment, we have provided more information (Manuscript – Tracked Changes (All Markup), page 8, section 2, lines 162-165) regarding potential discontinuation of the study intervention in the manuscript. The STF-intervention will be discontinued after review with the principal investigator if a subject experiences clinically significant side-effects necessitating caloric intake, f.i. hypoglycaemia.

11c can you give more detail on strategies to improve adherence by participants to protocols?

Thank you for this very relevant comment. Adherence to the protocol will be of critical importance to the outcome of our study. We will inform all enrolled patients of the importance of a reliable control and intervention arm and therefore the importance of adhering to their assigned diet. The informed consent also clearly states that participants must be adherent tot the protocol or may withdraw otherwise.

To improve adherence in the intervention arm, we will inform the patients in this arm of the importance of adhering to the fasting diet and providing them with preoperative information and clear instructions (both orally and in writing) regarding the diet. We will also make sure they do not receive the preoperative drink (current standard of care) before surgery, thereby making sure that they receive no caloric intake.

To improve adherence in the control arm, we will inform the patients in this arm of the importance of a reliable control arm and therefore the importance of adhering to their normal diet. We will also provide a preoperative drink, current standard of care, before surgery, thereby making sure that they receive caloric intake.

This advice is provided via the study coordinator, both orally and in writing, on two occasions (Manuscript – Tracked Changes (All Markup), page 8, section 2, lines 160-162).

11d Can you specify what concomitant treatments are allowed or prohibited?

Any non-experimental treatments are allowed if it does not have any nutritional (caloric) value. We have emphasized this in the revised manuscript (Manuscript – Tracked Changes (All Markup), page 8, section 2, lines 164-165).

16b Can you give more detail on the mechanism of implementing the allocation sequence

We have provided more info (Manuscript – Tracked Changes (All Markup), page 13, section 1, line 269) on the mechanism of randomization by stating the block sizes (4, 6 & 8) in the manuscript, used in the stratified block randomization procedure. This randomization procedure stratifies on both study centre (Erasmus & UMCG) and subject sex.

21b Will there be an interim analysis? What are the stopping guidelines?

There will be no interim analysis due to the relatively low number of patients needed and the patients that will be included are healthy, mentally capable, non-critical and not incapacitated.

Also, the short-term fasting diet used in this study is not known to harm the involved patients (Manuscript – Tracked Changes (All Markup), page 14, section 1, lines 300-302).

30 Is there any post-trial care or compensation for any who suffer harm during the trial?

Our apologies for an incorrect reference in the checklist: on page 13 we state that "potential compensation for any participant who suffers harm due to trial participation has been covered by the academic hospital insurance, in accordance with Dutch laws and regulations.". We have added additional information on post-trial care in the manuscript (Manuscript – Tracked Changes (All Markup), page 16, section 2, lines 360-363).

We again would like to thank both reviewers for their time and suggestions and hope that by making these changes we have met the requirements for publication. All authors have seen and approved the manuscript and are fully conversant with its contents.

Sincerely,

Drs. C.A.J. Oudmaijer
Corresponding Author
Erasmus MC Transplant Institute
University Medical Center Rotterdam
Dr. Molewaterplein 40
3015 GD Rotterdam
E-mail: c.oudmaijer@erasmusmc.nl
Tel: +316-50173127

Additional contact
Prof. dr. J.N.M. IJzermans
Erasmus MC Transplant Institute
University Medical Center Rotterdam
Dr. Molewaterplein 40
3015 GD Rotterdam
E-mail: j.ijzermans@erasmusmc.nl
Tel: +3110-7031149
